# Supplementary material for: Early onset diagnosis in Alzheimer’s disease patients via amyloid-β oligomers-sensing probe in cerebrospinal fluid
Source: Nat Commun. 2024 Feb 2;15:1004. doi: 10.1038/s41467-024-44818-x (PMC10837422; doi:10.1038/s41467-024-44818-x)
Supplement: Supplementary file 1 — Supplementary Information [file 41467_2024_44818_MOESM1_ESM.pdf]

Nature Communications
Supplementary Information

Early onset diagnosis in Alzheimer's disease patients via amyloid-β oligomers-sensing probe in cerebrospinal fluid

Jusung An,[a,†] Kyeonghwan Kim,[b,c,†] Ho Jae Lim,[d] Hye Yun Kim,[b,c] Jinwoo Shin,[a] InWook Park,[b,c] Illhwan Cho,[b,c] Hyeong Yun Kim,[b] Sunghoon Kim,[b,e,f] Catriona McLean,[g] Kyu Yeong Choi,[h] YoungSoo Kim,[b,c,]* Kun Ho Lee[d,h,i]* and Jong Seung Kim[a,j,]*

[a] Department of Chemistry, Korea University, Seoul 02841, Korea.

[b] Department of Pharmacy, College of Pharmacy, Yonsei University, Incheon 21983, Korea.

[c] Yonsei Institute of Pharmaceutical Sciences, College of Pharmacy, Yonsei University, Incheon 21983, Korea.

[d] Department of Biomedical Science, Chosun University, Gwangju 61452, Korea.

[e] Medicinal Bioconvergence Research Center, Institute for Artificial Intelligence and Biomedical Research, Gangnam Severance Hospital, Yonsei University, Incheon 21983, Korea.

[f] College of Pharmacy, College of Medicine, Interdisciplinary Biomedical Center, Gangnam Severance Hospital, Yonsei University, Incheon 21983, Korea.

[g] Department of Pathology, The Alfred Hospital, Melbourne 3004, Australia

[h] Gwangju Alzheimer's & Related Dementia Cohort Research Center, Chosun University, Gwangju 61452, Korea.

[i] Department of Neural Development and Disease, Korea Brain Research Institute, Daegu 41062, Korea.

[j] TheranoChem Incorporation, Seongbuk-gu, Seoul 02856, Korea.

[†] These authors contributed equally to this work.

Corresponding Author

Jong Seung Kim (jongskim@korea.ac.kr)

Kun Ho Lee (leekh@chosun.ac.kr)

YoungSoo Kim (y.kim@yonsei.ac.kr)

Supplementary Methods

General materials and instrumentation for synthetic studies and characterization

All chemical reagents and solvents used for synthesis were purchased from commercial suppliers (Sigma-Aldrich (Merck), Thermo-Fisher, TCI-Korea, Samchun, and Duksan) and used as received. Column chromatography was used with silica gel 100 (Merck) as a stationary phase for purification purposes of all synthetic compounds. To monitor the synthesis progress, thin layer chromatography (TLC) was used with Merck 60 F_{254} (pre-coated sheets, 0.25 mm thick) plates. Moisture-sensitive reactions were carried out under an inert atmosphere (high purity grade Argon gas), and anhydrous solvents were prepared with distilling filters. ^{1}H and ^{13}C nuclear magnetic resonance (NMR) spectral analysis was performed for the characterization of synthesized compounds on a 500 MHz Bruker NMR spectrometer at room temperature using deuterated chloroform-*d* ($CDCl_3$) with tetramethylsilane (TMS) as an internal standard (NMR spectra are presented by chemical shifts in ppm values and coupling constants in Hz). Mass spectroscopy (MS) was conducted on a Shimadzu LCMS-2020 electrospray ionization mass spectrometer (ESI-MS). UV/Vis spectra were recorded on a Jasco V-750 spectrometer, and fluorescence spectra were obtained using a Jasco FP-8500 spectrofluorometer. Circular dichroism spectra were measured on a Jasco J-1500 spectrometer at Korea Basic Science Institute (KBSI) Ochang Center in Korea. Electron microscopic imaging was recorded on a JEM-1400 Plus (Bio-High voltage EM, JEOL Ltd., Japan) at KBSI Ochang Center. Time course fluorescence intensity was recorded using a Hidex Sense Microplate Reader with a 96-well black plate (SPL, Korea) and adhesive optical sealing film (Bioneer Inc., Korea). Various organic solvents for photophysical analysis were used with high performance liquid chromatography (HPLC)-grade solvents purchased from J. T. Baker. Amyloid-β $(A\beta)_{1-42}$ was prepared by synthesizing and purchasing hexafluoroisopropanol (HFIP)-pretreated $A\beta_{1-42}$ (Human) peptides (purity = 95.47%), and $A\beta_{1-40}$ was prepared by purchasing HFIP-pretreated $A\beta_{1-40}$ (Human) peptides (purity > 95%) from GenicBio Synthetic Peptide (Shanghai, China). Artificial cerebrospinal fluid (aCSF) was purchased from Tocris Bioscience (Bristol, United Kingdom).

Compound synthesis

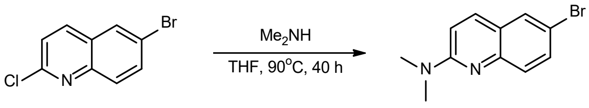

Synthesis of 6-bromo-*N,N*-dimethyl-2-quinolinamine (1). A pressure seal tube was equipped with 2-chloro-6-bromoquinoline (0.80 g) and Me_2NH (2 M in tetrahydrofuran (THF), 11 mL, 22 mmol) and stirred at 90°C for 40 h. The reaction mixture was cooled down to room temperature, subsequently quenched with H_2O and concentrated under reduced pressure. The residue was extracted using ethyl acetate (3 × 100 mL), washed with brine, and the organic layer was dried over anhydrous Na_2SO_4, filtered, and concentrated in vacuo. The crude was purified by silica gel column chromatography (ethyl acetate/hexane, 1:9) to afford compound 1 (yield: 92%).

Compound 1: ^{1}H NMR (500 MHz, $CDCl_3$) δ 7.58 (m, J = 1.3 Hz, 1H), 7.54 (d, J = 9.1 Hz, 1H), 7.51 (d, J = 1.5 Hz, 2H), 6.68 (d, J = 9.1 Hz, 1H), 3.09 (s, 6H) ppm. ^{13}C NMR (125 MHz, $CDCl_3$) δ 157.55, 146.92, 136.01, 132.40, 129.25, 128.15, 123.61, 114.09, 109.79, 37.96 ppm.

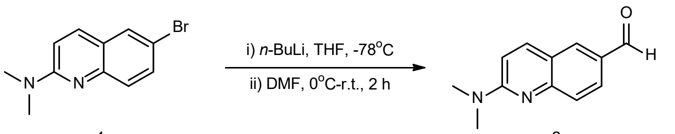

1 2

Synthesis of 2-(dimethylamino)-6-quinolinecarboxaldehyde (2). To a solution of compound 1 (0.50 g, 2.0 mmol) in anhydrous THF (5 mL) at −78°C under argon atmosphere was added *n*-butyl lithium (2.5 M in hexanes, 0.96 mL, 2.39 mmol). After the mixture was stirred for 30 min, *N,N*-dimethylformamide (DMF) was added, and the mixture was stirred for a further 2 h at 0°C. The reaction mixture was quenched by the dropwise addition of saturated NH_4Cl solution (10 mL) over 10 min. Next, the reaction mixture was extracted with ethyl acetate (3 × 100 mL), and the combined organic layers were dried over anhydrous Na_2SO_4, filtered, and concentrated under reduced pressure. The residue was purified by silica gel column chromatography (ethyl acetate/hexane, 1:3) to afford compound 2 (yield: 88%).

Compound 2: ^{1}H NMR (500 MHz, $CDCl_3$) δ 10.01 (s, 1H), 8.07 (d, J = 1.9 Hz, 1H), 8.00 (dd, J = 8.7, 1.9 Hz, 1H), 7.94 (d, J = 9.1 Hz, 1H), 7.71 (d, J = 8.7 Hz, 1H), 6.94 (d, J = 9.2 Hz, 1H), 3.28 (s, 6H) ppm. ^{13}C NMR (125 MHz, $CDCl_3$) δ 191.29, 158.80, 152.11, 138.12, 133.22, 130.08, 127.57, 127.06, 121.44, 109.86, 37.96 ppm.

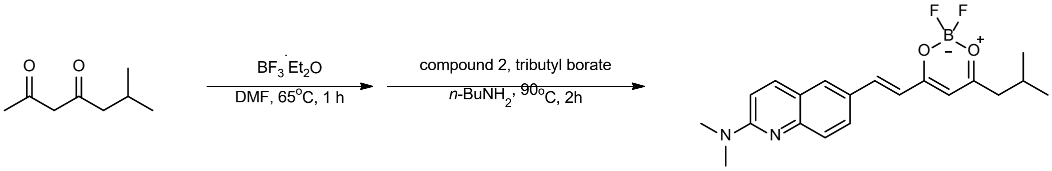

Q-OB

Synthesis of (*E*)-4-(2-(2-(dimethylamino)quinolin-6-yl)vinyl)-2,2-difluoro-6-isobutyl-2*H*-1,3,2-dioxaborinin-1-ium-2-uide (Q-OB). To a solution of 6-methylheptane-2,4-dione (0.041 mL, 0.26 mmol) in anhydrous DMF (0.5 mL) at 65°C under argon atmosphere was added boron trifluoride etherate (0.049 mL, 0.40 mmol). After the reaction mixture was stirred for 30 min, compound 2 (50 mg, 0.26 mmol), tributyl borate (0.14 mL, 0.53 mmol), and *n*-$BuNH_2$ (20 mol %) were added and then stirred at 90°C for 4 h in situ. The reaction mixture was cooled down to room temperature and was extracted with ethyl acetate (3 × 50 mL), and the combined organic layers were dried over anhydrous Na_2SO_4, filtered, and concentrated in vacuo. The residue was purified by silica gel column chromatography (ethyl acetate/hexane, 1:2) to afford compound Q-OB (yield: 52%).

Q-OB: ^{1}H NMR (500 MHz, $CDCl_3$) δ 8.17 (d, J = 15.4 Hz, 1H), 7.86 (d, J = 9.2 Hz, 1H), 7.79–7.75 (m, 2H), 7.65 (d, J = 9.4 Hz, 1H), 6.91 (d, J = 9.2 Hz, 1H), 6.63 (d, J = 15.4 Hz, 1H), 5.95 (s, 1H), 3.27 (s, 6H), 2.40 (d, J = 7.2 Hz, 2H), 2.30–2.15 (sept, 1H), 1.01 (d, J = 6.6 Hz, 6H) ppm. ^{13}C NMR (125 MHz, $CDCl_3$) δ 192.78, 180.54, 158.53, 150.99, 148.85, 137.80, 132.15, 127.78, 127.40, 127.05, 122.17, 117.48, 109.90, 101.41, 46.60, 38.06, 27.27, 22.51 ppm. ESI-MS: $C_{20}H_{23}BF_2N_2O_2$ $[M+H]^+$, *m/z* calcd 373.2246, found 373.15; $[M–H]^-$, *m/z* calcd 371.2092, found 371.05; $[M–BF_2+H]^+$ *m/z* calcd 325.4242, found 325.10.

Quantum yield and determination of fluorescence emission and wavelength maxima

To avoid the inner filter effect during the measurement of fluorescence emission, the spectra were recorded with an absorbance lower than 0.1 at wavelengths longer or equal to the excitation wavelength with HPLC grade solvents, including 1,2-dichlorobenzene, 1-butanol, 1-propanol, 2-methoxy ethanol, acetone, acetonitrile, chloroform, diethyl ether, ethyl acetate, ethylene glycol dimethyl ether, ethanol, methanol, THF, or toluene). The fluorescence quantum yield of Q-OB was measured versus 4-(dicyanomethylene)-2-methyl-6-(4-dimethylaminostyryl)-4*H*-pyran (DCM) in acetonitrile (Φ_{FL} = 0.43)[1], using a previously reported procedure[2].

Spectroscopy in a solvent with increasing viscosity

The fluorescence emission spectra of Q-OB and Thioflavin T (ThT) (10 μM) in the various v/v % of ethylene glycol and glycerol (30, 50, 60, 70, and 80% of glycerol in ethylene glycol) at 25°C were recorded. Q-OB and ThT stock solutions were prepared in dimethyl sulfoxide (DMSO), respectively, and all final solutions contained a final concentration of 1% DMSO.

Solubility of Q-OB in aqueous solution

The absorbance of Q-OB was recorded at 460 nm in phosphate-buffered saline (PBS) (10 mM, pH = 7.4) containing 1% DMSO at various concentrations in the 0−50 μM range.

pH dependent absorbance of Q-OB

The absorbance and fluorescence spectra of Q-OB (10 μM) were recorded in deionized water (DW) containing 1% DMSO with the pH adjusted by hydrochloric acid or sodium hydroxide to the pH 1 to 11.

Photostability

A solution of Q-OB with an absorbance of 1.0 at the maximum absorbance wavelength was prepared in DMSO and 1% DMSO/PBS (10 mM, pH = 7.4) in the presence of A$\beta_{1\text{–}42}$ (10 μM). A 3100 K halogen lamp (Olympus LG-PS2; 12 V, 100 W) was used for light irradiation (irradiation distance = 30 cm), and the absorbance was recorded at 1 h intervals for 12 h.

Cross-reactivity

The fluorescence intensity of Q-OB (1 μM) was recorded with A$\beta_{1\text{–}42}$ oligomers (0.1 μM) in the presence of cross-reactive interferents, including A$\beta_{1\text{–}40}$ monomer, A$\beta_{1\text{–}42}$ monomer, A$\beta_{1\text{–}40}$ fibrils, or A$\beta_{1\text{–}42}$ fibrils, prepared in various concentration ratios for A$\beta_{1\text{–}42}$ oligomers (×0 (blank), ×1, ×10, ×100, and ×500) in PBS (10 mM, pH = 7.4) (λ_{ex} = 460 nm, slit 2.5/2.5, n = 3 independent experiments).

Density functional theory calculations

Structure optimization of Q-OB was performed using the Gaussian 16 software package[3], at the ωB97XD/N07D level of theory using the default integral equation formalism variant of the polarizable continuum model (IEFPCM) solvation model of acetonitrile[4,5]. The molecular orbital was visualized using Gaussview 6.1. To determine the amount of intramolecular charge transfer (ICT) character upon vertical excitation to the first excited state, a calculation based on the differences in electron density was used[6], and results were analyzed using Multiwfn 3.8[7]. For these calculations, the molecular structure was recalculated at the CAM-B3LYP/N07D level of theory[8], using the IEFPCM model of water, and time-dependent density functional theory (TDDFT) vertical excitations were performed using the same solvation model, and the optimized structure of the ground state.

Circular dichroism spectroscopy

Circular dichroism measurements of A$\beta_{1\text{–}42}$-species were conducted in PBS (10 mM, pH = 7.4). The concentration of the protein sample was 10 μM and the circular dichroism spectra were measured from 200 to 260 nm using a 1 mm path length quartz cuvette with a spectral bandwidth of 1 nm, a signal averaging time of 1 s, and a data interval of 0.5 nm at

37°C. The spectra presented are the average of three independent experiments and corrected using a control solution lacking Aβ.

$A\beta_{1–40}$ in vitro kinetics assay

Prepared 46.2 mL $A\beta_{1–40}$ DMSO stock solution (5 mM) was diluted with 4.6 mL PBS (20 mM, pH = 7.4) (working concentration was 50 μM of $A\beta_{1–40}$ containing 1% DMSO). Eight set of the diluted solution was incubated at an orbital shaker (37°C, 300 rpm), and 2970 μL was collected every 12 h for 7 days. 30 μL of Q-OB DMSO solution (100 μM) (or 1 mM of ThT DMSO solution) was added to the collected PBS, and fluorescence spectra were measured (λ_{ex} = 460 nm for Q-OB and λ_{ex} = 440 nm for ThT, respectively) (slit 2.5/2.5, *n* = 3 independent experiments).

Cell culture

Human-derived neuroblastoma cell line SH-SY5Y was grown on 100 mm culture dishes and maintained in 5% (v/v) CO_2 and 95% (v/v) humidity at 37°C. The cell was cultured in Dulbecco's Modified Eagle Medium/Nutrient Mixture F-12 (DMEM/F12) medium (Gibco), containing 10% (v/v) fetal bovine serum (FBS) (HyClone) (100 units/mL) and streptomycin-penicillin (HyClone) (100 μg/mL). When the culture dishes reached 70% confluence, the cells were sub-cultured with 0.05% trypsin-ethylenediaminetetraacetic acid (EDTA) and seeded on the new 100 mm cell culture dishes.

Cell viability

SH-SY5Y cell was seeded on the 96-well plates at 2.0×10^4 cells/well separately and allowed to adhere to the plate for 48 h. Then the cells were treated with Q-OB (0, 0.1, 1, 5, 10, 25, 50, and 100 μM) and incubated for 24 h. Remove the medium, followed by washing each well with PBS (10 mM, pH = 7.4). After irradiation using a 488 nm lamp (100 mW/cm^2, 1 min; 6 J/cm^2), the viability was assessed using a water-soluble tetrazolium (WST-8)-based cell viability assay kit (Cellomax, CM-VA2500) for detecting the NAD(P)H in the mitochondria of living cells with/without photo-irradiation conditions. Incubated 2 h with the WST-8 solution by adjusting the WST-8 solution volume in a well to 10% of the total volume. Then measured the absorbance at 450 nm using a microplate reader.

Reactive oxygen species generation

The generation of reactive oxygen species (ROS) by Q-OB was measured using 1,3-diphenylisobenzofuan (DPBF) as a ROS indicator with the indocyanine green (ICG), methylene blue (MB), and rose bengal (RB) as a reference compound. 3 mL DMSO mixture of DPBF (50 μM) and Q-OB (or each photosensitizer) (5 μM) was prepared into cuvette; then the cuvette was irradiated using Xe-lamp up to 5 min (λ_{ex} = 488 nm for Q-OB, 530 nm for RB, 660 nm for MB, and 800 nm for ICG, respectively) (1 mW/cm^2). The absorption spectra were recorded immediately after each irradiation period.

Parallel artificial membrane permeability assay

Parallel artificial membrane permeability assay (PAMPA)-blood-brain barrier (BBB) studies were performed using the PAMPA-BBB kit (BioAssay Systems, PAMPA-096) according to the manufacturer's instructions. 5 mM of the Q-OB and controls stock solutions were prepared in DMSO, which were then diluted with PBS (10 mM, pH = 7.4) to a final concentration of 250 μM. The mimics cell membranes on each donor plate well were coated by adding 5 μL of the 4% Lecithin. Then, 200 μL of the Q-OB and controls were added to each well of the donor plate, and each well of the acceptor plate was filled with 300 μL of PBS. The donor plate was placed on the acceptor plate to form a sandwich, and

in separate tubes prepared equilibrium standards for Q-OB and controls; the sandwich and equilibrium standards were incubated at 37°C for 18 h without stirring. 100 μL of equilibrium standard, acceptor, and donor solutions were transferred to UV plates, and the absorbance spectrum of the solutions was measured from 200 to 500 nm in 10 nm intervals using a plate reader (SpectraMax M2e). Effective permeability (P_e) for samples was calculated using the data analysis equation specified in the manufacturer's instructions of BioAssay Systems. High permeability control (promazine hydrochloride), medium permeability control (clonidine), and low permeability control (diclofenac) were utilized as positive controls and negative controls for this assay. Based on their measured effective permeability $-\log P_e$, compounds are categorized as either BBB permeable (CNS+) or BBB impermeable (CNS–). Compounds were classified as CNS+ if their $-\log P_e$ value is below the low permeability control $-\log P_e$ value.

Organ distribution ex vivo imaging

To observe the distribution of Q-OB in mice, 200 μL Q-OB was intravenously injected to 2-month-old wild-type B6 mice. After 1 h, isoflurane was given to the mice through inhalation, and they were then sacrificed after being fully anesthetized. Their brain, liver, heart, and kidneys were extracted and washed with saline to remove blood from the surfaces. The prepared organs were located on the stage in the IVIS® Spectrum In Vivo Imaging System (Perkin-Elmer, USA) and fluorescence intensity of Q-OB was measured ($\lambda_{ex}/\lambda_{em}$ = 500/620 nm).

Supplementary Tables and Figures

Supplementary Table 1. Reported small-molecule fluorescent probes for amyloid-β (Aβ) oligomers.

Probe name	Chemical structure	Max. $\lambda_{abs}/\lambda_{em}$ (nm)	K_D (nmol/L) for Aβ species	$I_{oA\beta}/I_{mA\beta}$	$I_{oA\beta}/I_{iA\beta}$	Target oligomer	Potential clinical application	Ref.
BD-Oligo	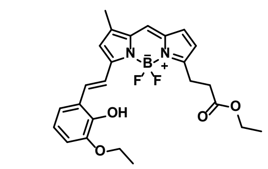	530/585	480 ($A\beta_{40}$ oligomer)	~4.0-folds	~2.6-folds	$A\beta_{40}$ Trimer (putative)	-	9
CRANAD-3	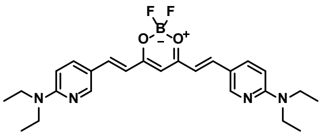	600/730	23 ± 5.7 ($A\beta_{40}$ monomer); 21 ± 2.3 ($A\beta_{40}$ fibril); 24 ± 1.6 ($A\beta_{42}$ monomer); 16 ± 6.7 ($A\beta_{42}$ dimer); 27 ± 15.8 ($A\beta_{42}$ oligomer)	~0.55-folds	~0.88-folds	undefined $A\beta_{42}$ oligomer	-	10
CRANAD-58	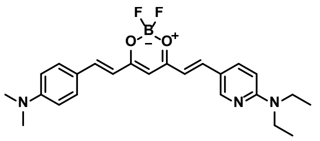	~630/ ~700	105.8 ($A\beta_{40}$ fibril); 45.8 ($A\beta_{42}$ fibril)	n.d.	n.d.	$A\beta_{40}$ dimer and undefined oligomer	-	11
CRANAD-102	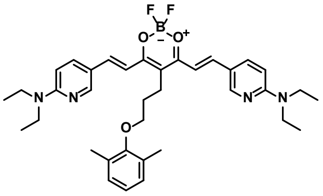	~580/ ~750	7.5 ± 10.0 ($A\beta_{42}$ oligomer); 505.9 ± 275.9 ($A\beta_{42}$ fibril);	~0.66-folds	1.2-folds	$A\beta_{42}$ dimer and undefined oligomer	-	12
F-CRANAD-101	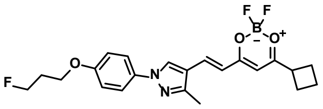	~420/ ~550	592.3 ± 123.2 ($A\beta_{42}$ oligomer) 188.5 ± 104.4 ($A\beta_{42}$ fibril)	~2.1-folds	n.d.	$A\beta_{42}$ dimer and undefined $A\beta_{42}$ oligomer	PET imaging	13
PTO-29	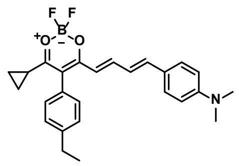	570/660	2703 ± 639 ($A\beta_{42}$ oligomer) 248 ± 48 ($A\beta_{42}$ fibril);	~4.2-folds	~3.8-folds	$A\beta_{42}$ Trimer (putative)	-	14
AD-1	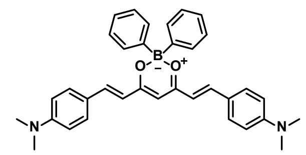	550/ ~650	1040 ± 335.2 ($A\beta_{42}$ monomer); 769.4 ± 221.5 ($A\beta_{42}$ oligomer); 356.1 ± 112.7 ($A\beta_{42}$ fibril)	~1.1-folds	~2.0-folds	$A\beta_{42}$ Trimer (putative)	-	15
AN-SP	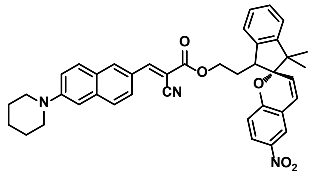	430/557	1700 ($A\beta_{42}$ oligomer)	~3.8-folds	~10.1-folds	undefined $A\beta_{42}$ oligomer	-	16

F-SLOH	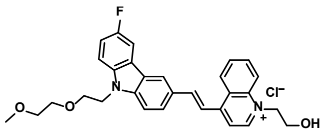	488/620	3220 ($A\beta_{40}$ monomer); 660 ($A\beta_{40}$ oligomer); 1900 ($A\beta_{40}$ fibril)	~5.0-folds	~2.5-folds	$A\beta_{40}$ Trimer (putative)	-	17
DCM-AN	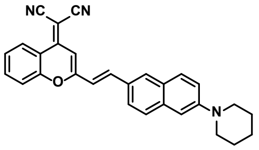	~500/ ~650	850 ($A\beta_{42}$ oligomer)	~3.7-folds	~3.7-folds	$A\beta_{42}$ Trimer (putative)	-	18
Eth-BF	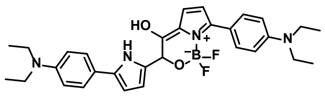	850/ 1015	6.16 ± 2.18 ($A\beta_{42}$ oligomer); 58.68 ± 36.2 ($A\beta_{42}$ fibril)	n.d.	~2.6-folds	$A\beta_{42}$ Trimer	-	19
Aza-BODIPY	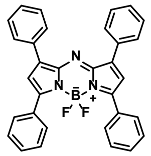	~600/ ~670	n.d.	n.d.	~2.6-folds	undefined $A\beta_{42}$ oligomer	-	20
LS-4	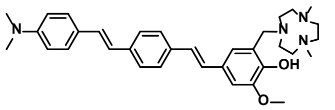	~380/ ~475	50 ± 9 ($A\beta_{42}$ oligomer); 58 ±15 ($A\beta_{42}$ fibril)	n.d.	~2.0-folds	$A\beta_{42}$ oligomer embedded in a lipid bilayer (putative)	PET imaging	21
**Q-OB**	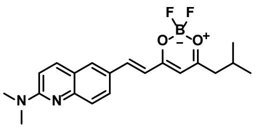	**460/580**	**4745.18 ± 722.36 ($A\beta_{42}$ monomer); 302.63 ± 94.75 ($A\beta_{42}$ oligomer); 5667.33 ± 1801.53 ($A\beta_{42}$ aggregate)**	**4.38-folds**	**2.51-folds**	**100 kDa over $A\beta_{42}$ oligomers**	**In vitro early onset diagnosis of AD**	-

Data were obtained from the reported references. The abbreviated words are amyloid-β (Aβ), Aβ monomers (*m*Aβ), Aβ oligomers (*o*Aβ), insoluble Aβ fibril (*i*Aβ), Alzheimer's disease (AD), positron emission tomography (PET), and K_D value indicates dissociation constant. Not available data marked as n.d. Additional information is available in the reported article[22].

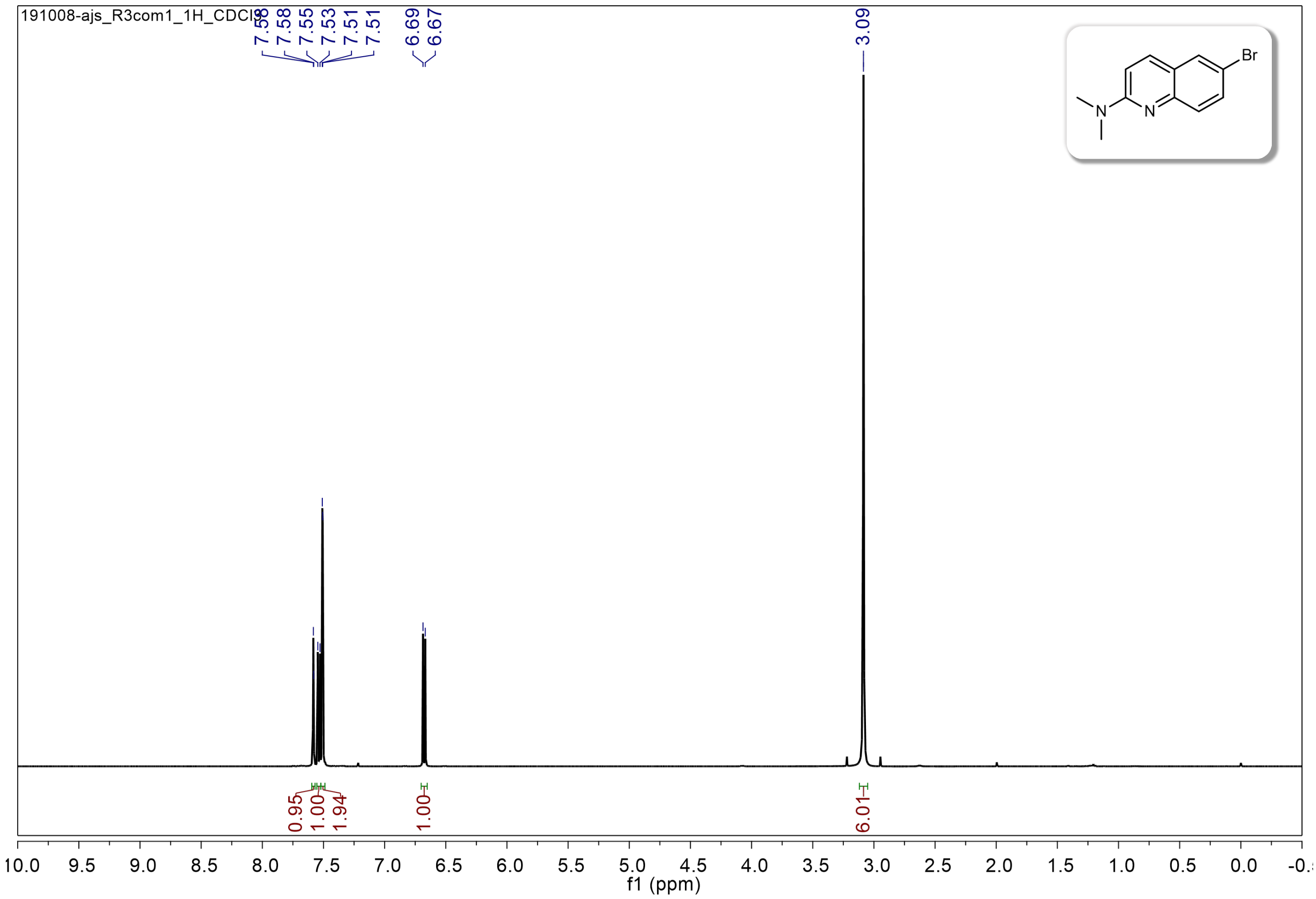

Supplementary Figure 1. ^{1}H NMR spectrum (500 MHz) of compound 1 in $CDCl_3$.

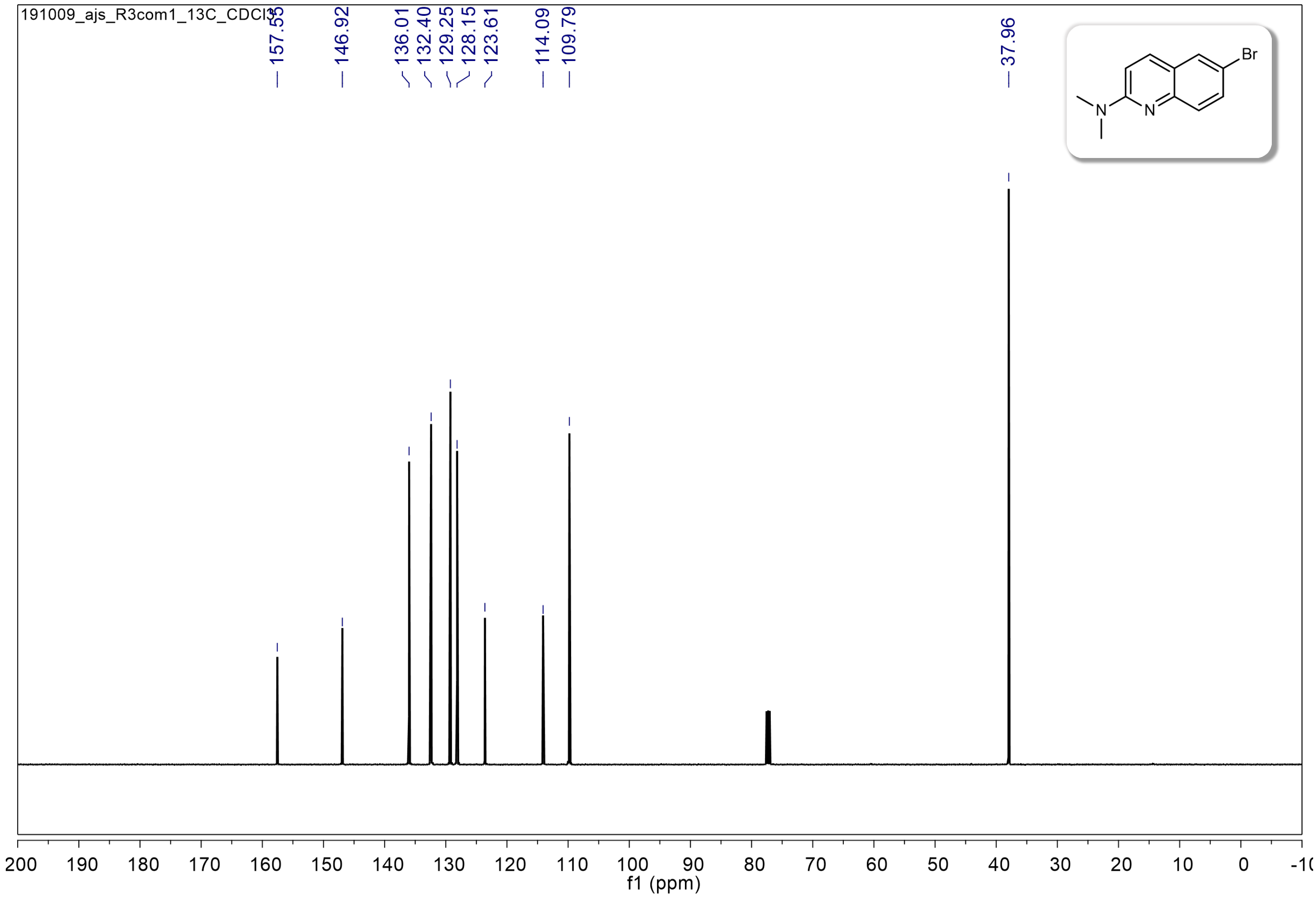

Supplementary Figure 2. ^{13}C NMR spectrum (125 MHz) of compound 1 in $CDCl_3$.

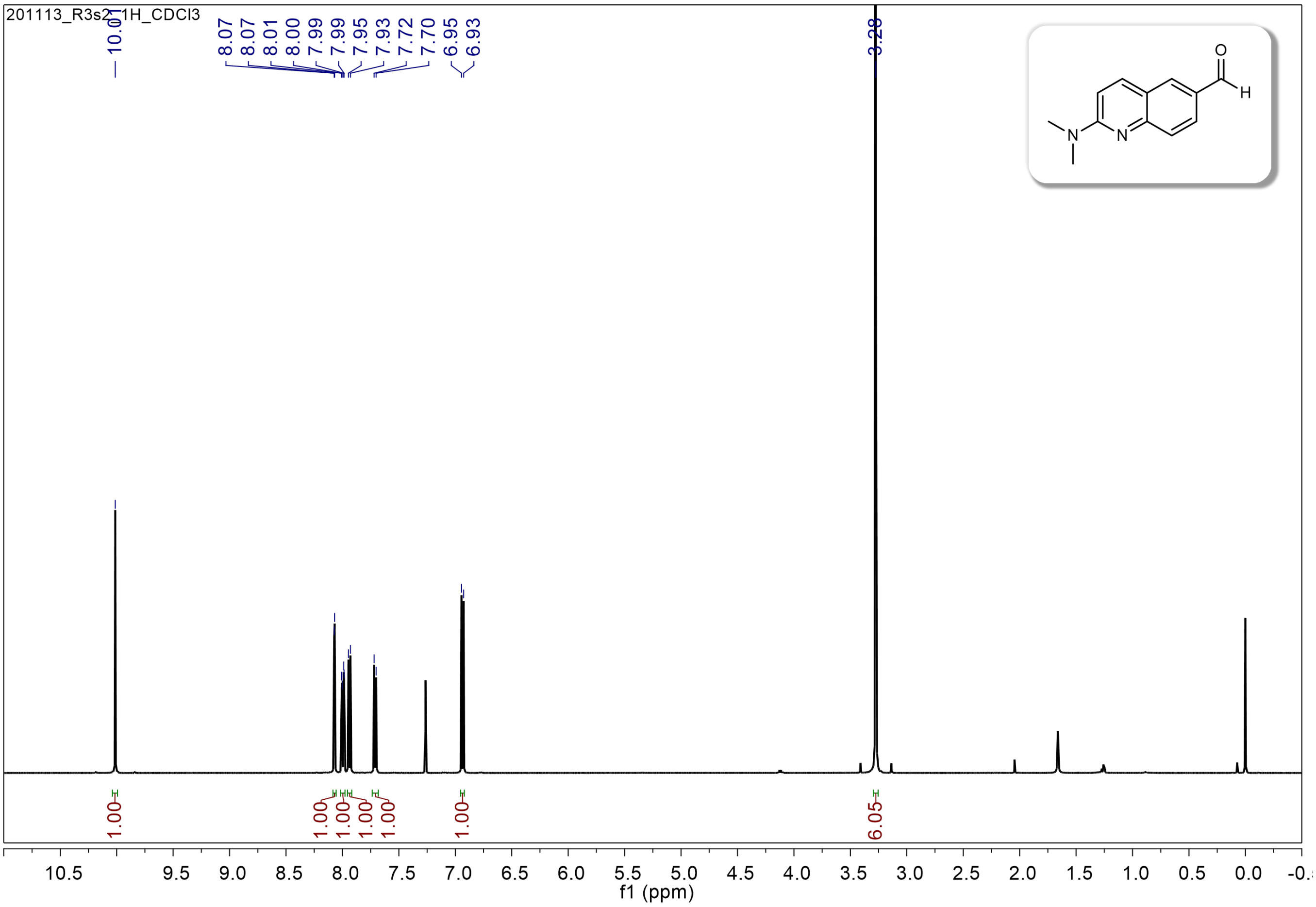

Supplementary Figure 3. ^{1}H NMR spectrum (500 MHz) of compound 2 in $CDCl_3$.

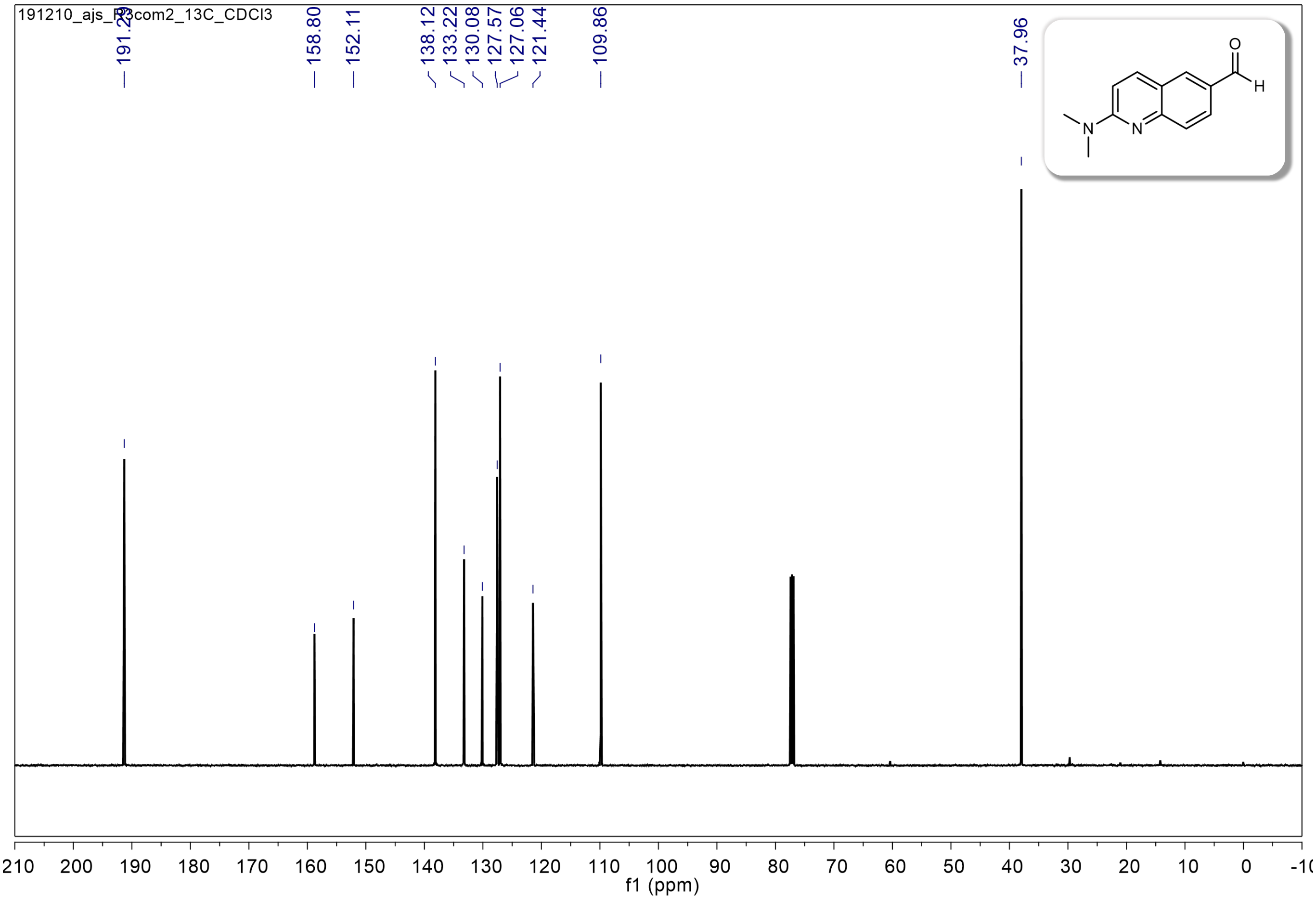

Supplementary Figure 4. ^{13}C NMR spectrum (125 MHz) of compound 2 in $CDCl_3$.

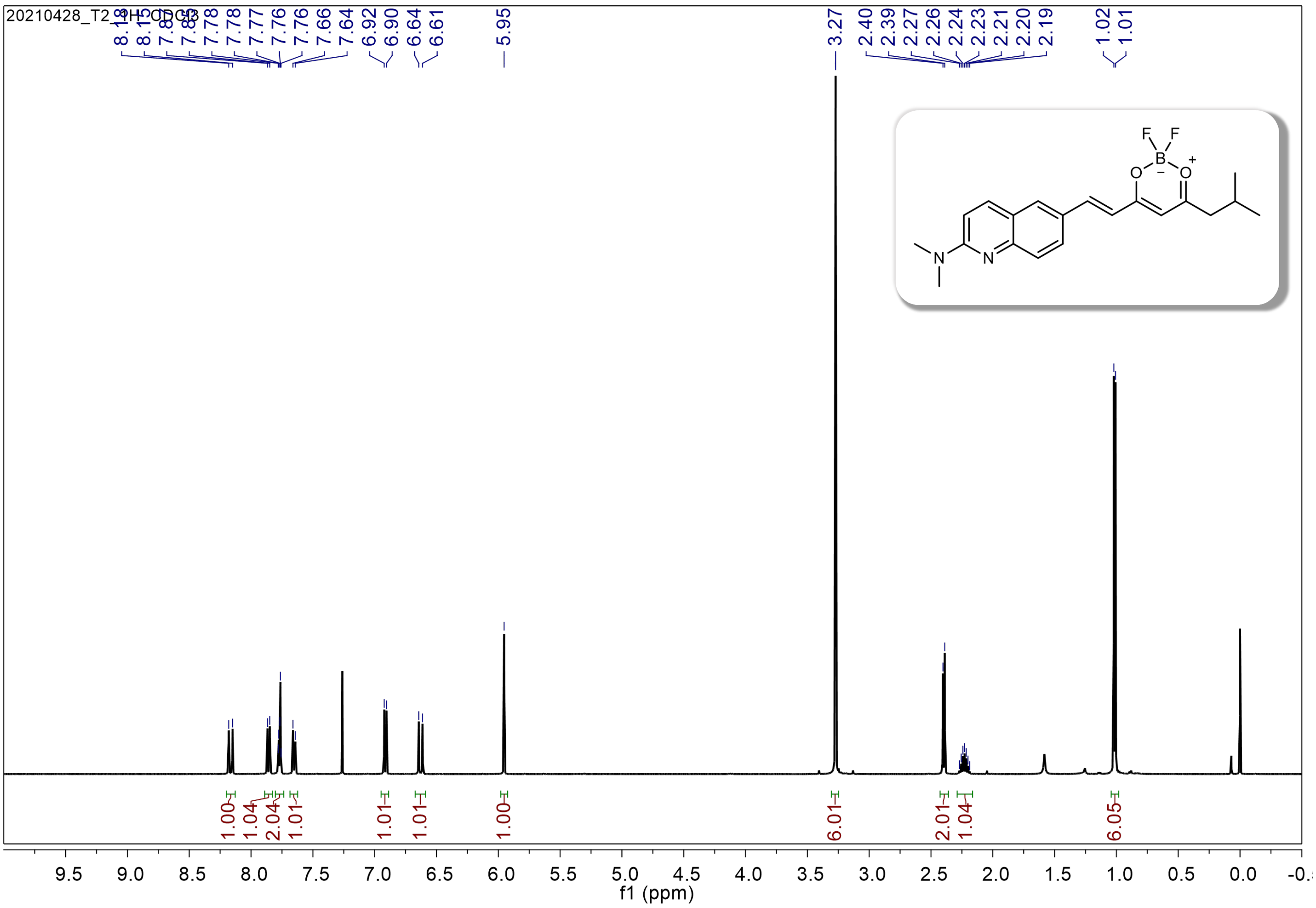

Supplementary Figure 5. ^{1}H NMR spectrum (500 MHz) of Q-OB in $CDCl_3$.

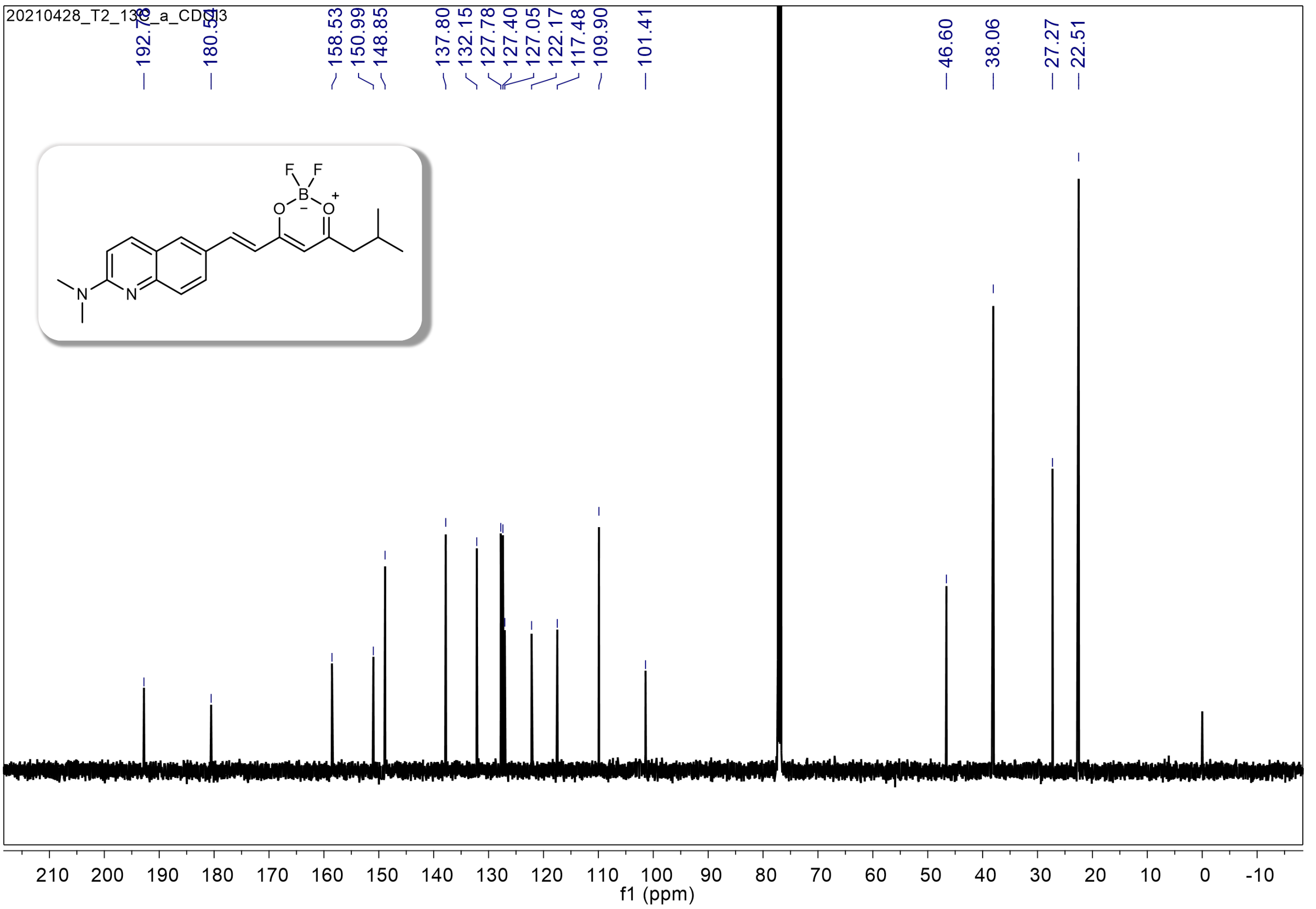

Supplementary Figure 6. ^{13}C NMR spectrum (125 MHz) of Q-OB in $CDCl_3$.

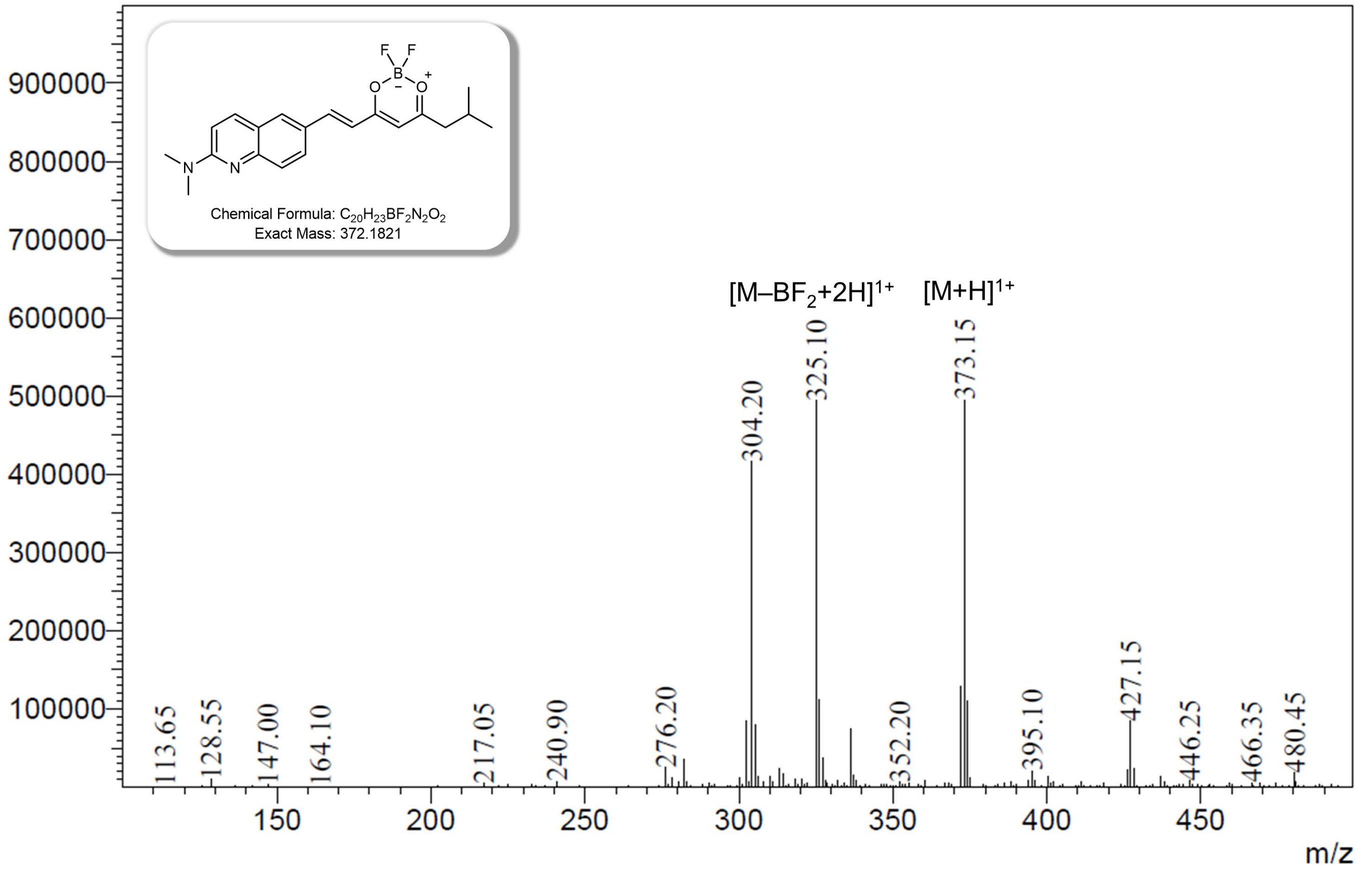

Supplementary Figure 7. ESI-MS spectrum (positive range) of Q-OB.

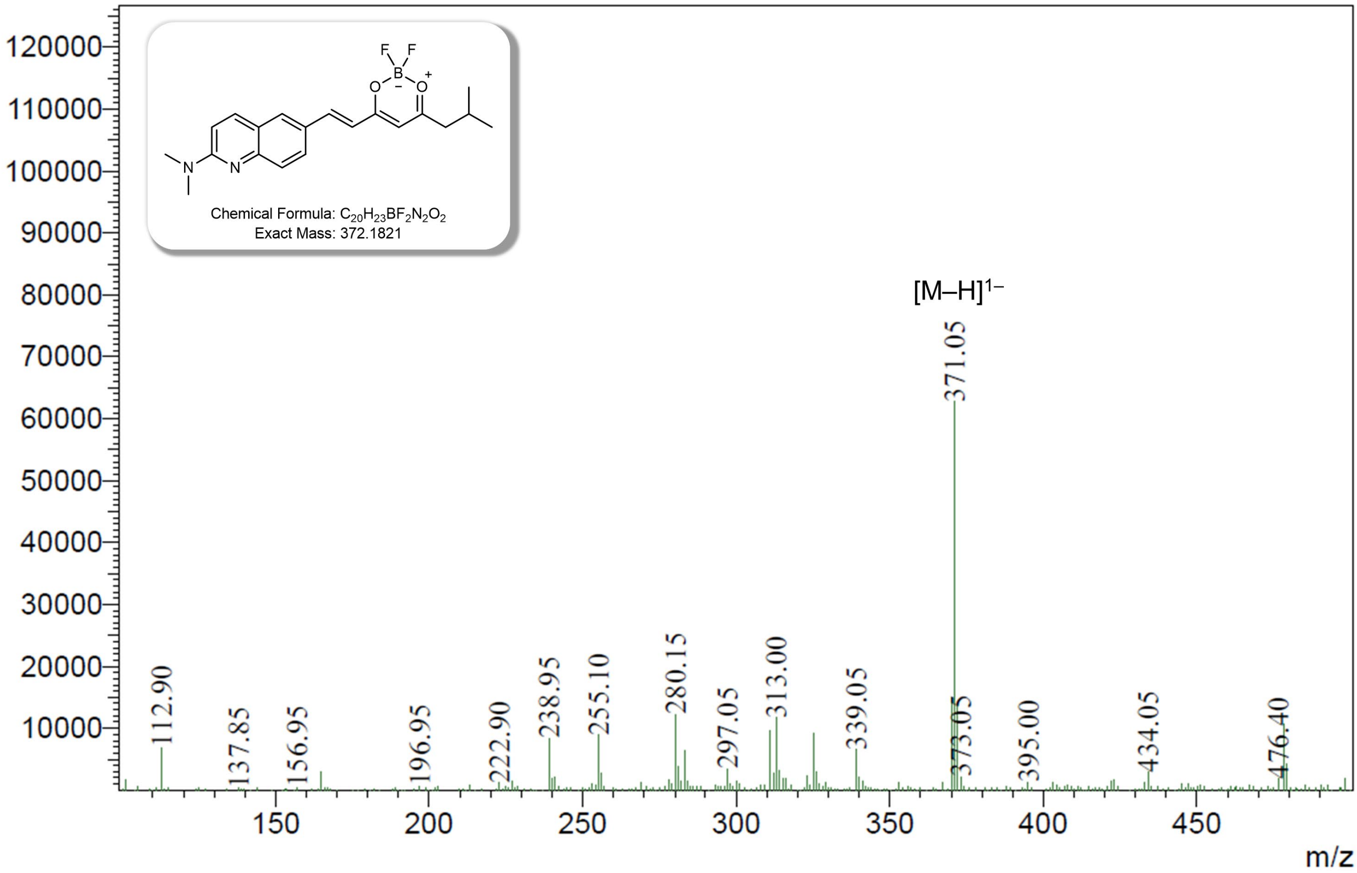

Supplementary Figure 8. ESI-MS spectrum (negative range) of Q-OB.

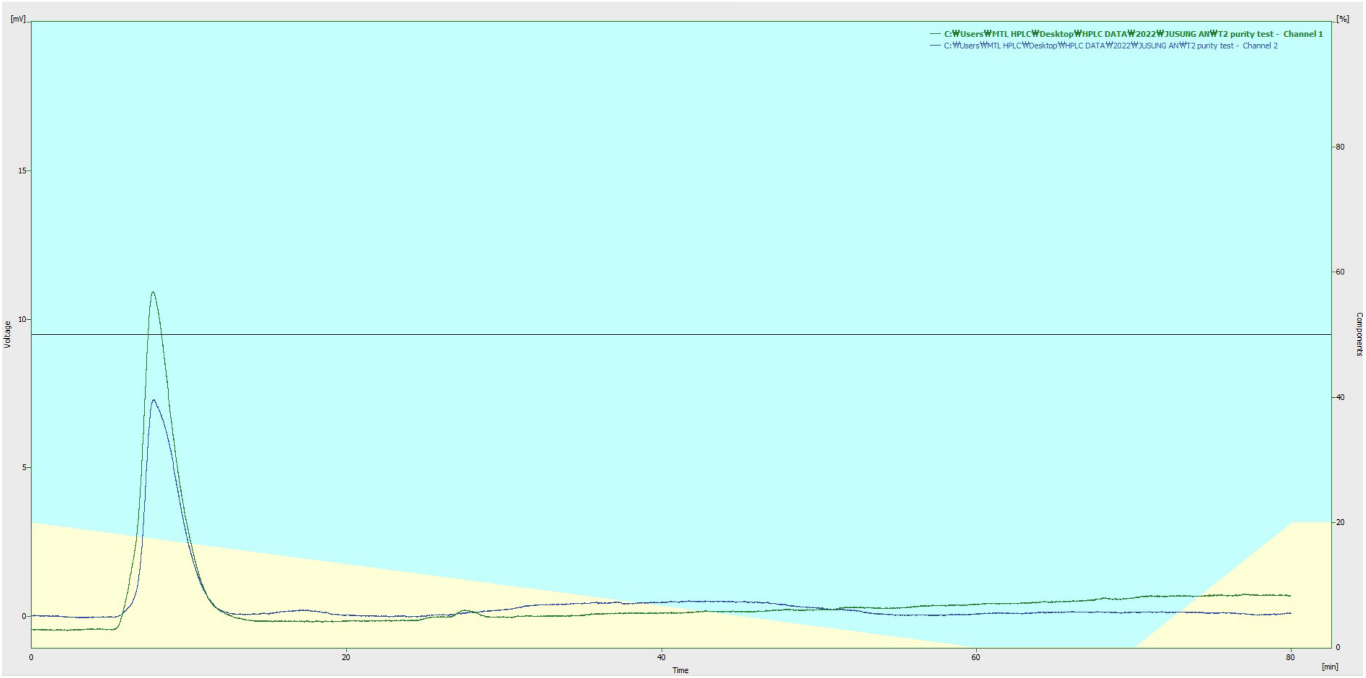

Supplementary Figure 9. HPLC spectrum of Q-OB.

Supplementary Table 2. Photophysical properties for Q-OB in various organic solvents.

Solvents	Q-OB				Solvent viscosity (cP)	Solvent dielectric constant	Solvent polarizability	Solvent dipolarity
	λ_{Abs} (nm)	λ_{Em} (nm)	$\Delta\lambda$ (nm)	ϕ_{Fl} (%)				
1,2-Dichlorobenzene	482	584	102	84.1	1.32	9.93	0.869	0.676
1-Butanol	456	604	148	71.0	2.95	17.8	0.783	0.655
1-Propanol	455	612	157	47.2	2.26	20.1	0.658	0.748
2-Methoxy ethanol	462	618	156	47.5	1.72	16.9	n.d.	n.d.
Acetone	460	609	149	81.7	0.32	20.7	0.651	0.907
Acetonitrile	462	630	168	17.1	0.37	37.5	0.645	0.974
Chloroform	470	569	99	72.0	0.58	4.81	0.674	0.614
Diethyl ether	460	543	83	70.6	0.24	4.34	0.617	0.385
Ethyl Acetate	457	573	116	66.7	0.46	6.02	0.656	0.603
Ethylene glycol dimethyl ether	461	585	124	51.1	0.46	7.2	0.68	0.625
EtOH	456	615	159	29.6	1.1	24.55	0.633	0.783
MeOH	450	624	174	8.2	0.55	32.6	0.608	0.904
THF	461	577	116	91.8	0.55	7.6	0.714	0.634
Toluene	469	534	65	79.7	0.59	2.4	0.782	0.284

Fluorescence quantum yields were determined vs. 4-(dicyanomethylene)-2-methyl-6-(4-dimethylaminostyryl)-4*H*-pyran (DCM) in acetonitrile[1]. Source data are provided as a Source Data file.

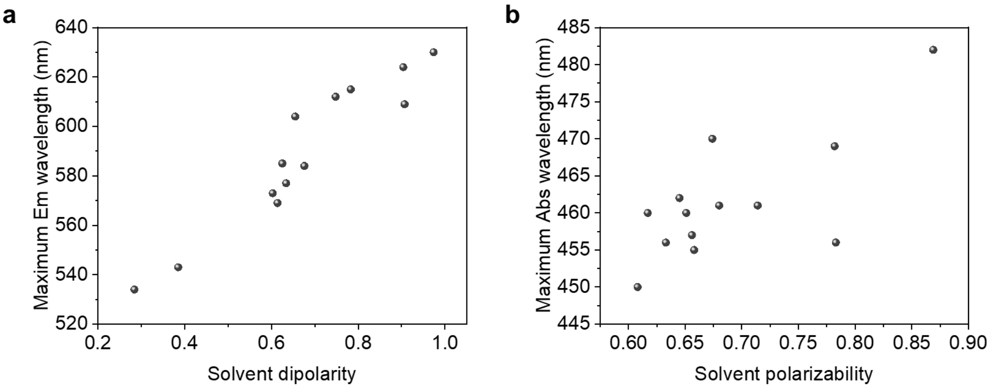

Supplementary Figure 10. Photophysical properties as an intramolecular charge transfer (ICT). **a** The relationship between the maximum absorbance wavelength of Q-OB plotted versus the solvent polarizability parameters and (**b**) The relationship between the maximum emission wavelength of Q-OB plotted versus the solvent dipolarity parameters. The various solvent (1,2-dichlorobenzene, 1-butanol, 1-propanol, acetone, acetonitrile, chloroform, diethyl ether, ethyl acetate, ethylene glycol dimethyl ether, ethanol, methanol, tetrahydrofuran, and toluene) referred to the Catalán dataset. The abbreviated words are Em, emission; Abs, absorbance. Source data are provided as a Source Data file.

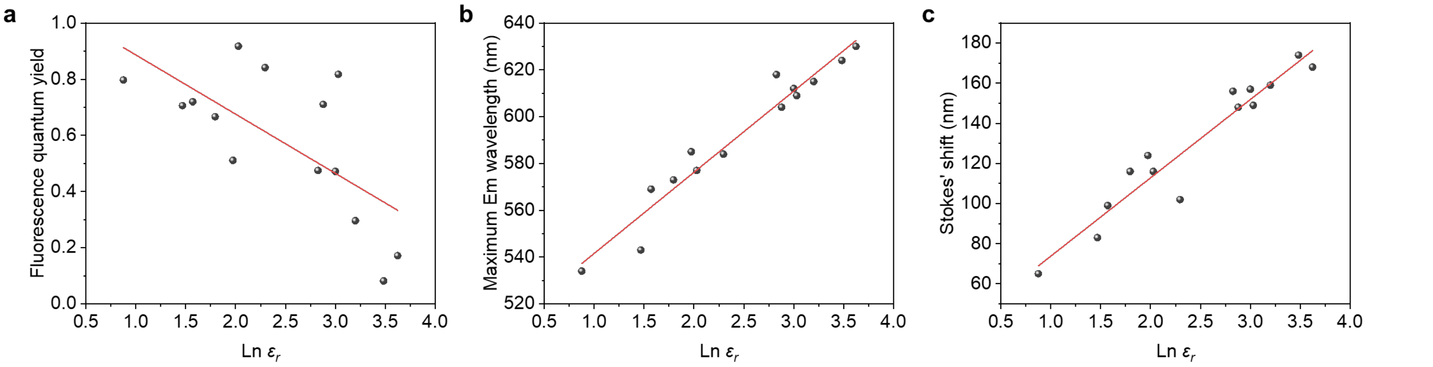

Supplementary Figure 11. The relation between the natural logarithm of the solvent dielectric constant and the (**a**) fluorescence quantum yield, (**b**) maximum emission wavelength, or (**c**) Stokes' shift of Q-OB with various solvents (1,2-dichlorobenzene, 1-butanol, 1-propanol, 2-methoxy ethanol, acetone, acetonitrile, chloroform, diethyl ether, ethyl acetate, ethylene glycol dimethyl ether, ethanol, methanol, tetrahydrofuran, or toluene). Source data are provided as a Source Data file.

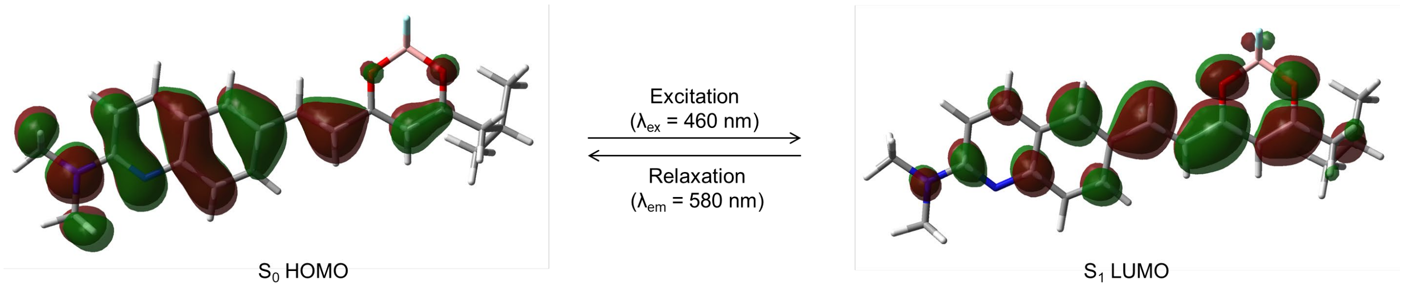

Supplementary Figure 12. Calculated molecular frontier orbitals of Q-OB. The abbreviated words are HOMO, highest occupied molecular orbital; LUMO, lowest unoccupied molecular orbital.

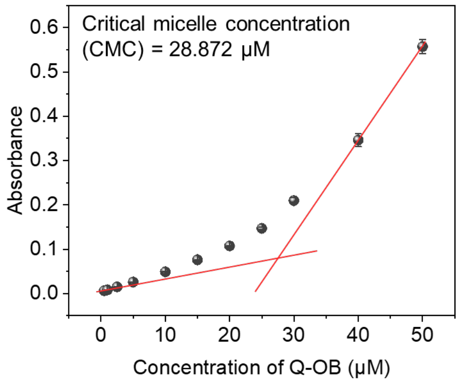

Supplementary Figure 13. Absorbance of Q-OB at various concentrations (0−50 μM) in phosphate-buffered saline (PBS) (10 mM, pH = 7.4) containing 1% dimethyl sulfoxide (DMSO). A critical micelle concentration (CMC) was observed for Q-OB concentrations at 28.872 μM. Error bars indicate standard deviation (s.d.), $n = 3$ independent experiments. Source data are provided as a Source Data file.

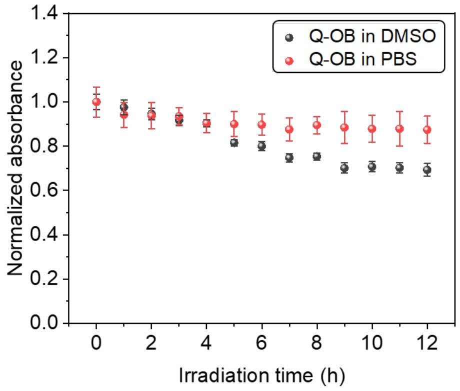

Supplementary Figure 14. Photostability of Q-OB in DMSO and 10 μM A$\beta_{1–42}$ in PBS (containing 1% DMSO). A 3100 K halogen lamp (Olympus LG-PS2, 12 V, 100 W) was used for irradiation, and the absorbance was recorded for 12 h (intervals: 1 h). The normalized absorbance changes were plotted. Error bars indicate s.d. derived from $n = 3$ independent experiments. Source data are provided as a Source Data file.

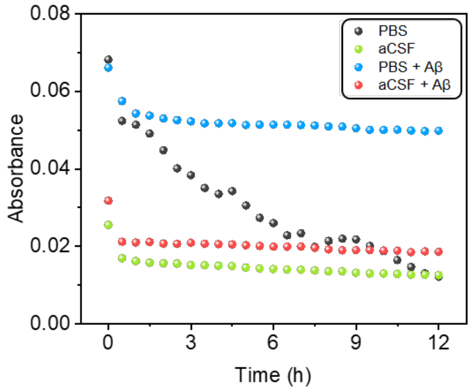

Supplementary Figure 15. Absorbance changes of Q-OB in PBS (10 mM, pH = 7.4) or artificial cerebrospinal fluid (aCSF) containing 10 μM Aβ (1% DMSO). Source data are provided as a Source Data file.

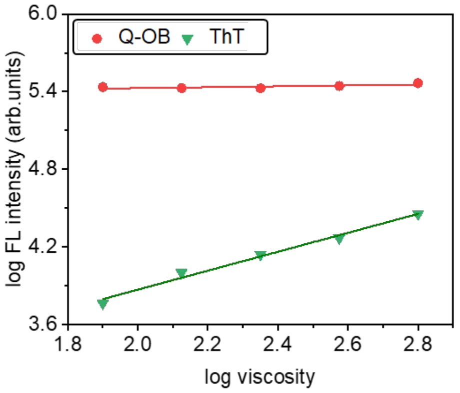

Supplementary Figure 16. Viscosity sensitivity of Q-OB and thioflavin T (ThT). Viscosity sensitivity calculated based on the fluorescence intensity in ethylene glycol and glycerol mixture. Experimental procedures followed the reported methods[23]. Source data are provided as a Source Data file.

Supplementary Table 3. Photophysical properties of fluorophores measured in glycerol containing 1% DMSO.

Compounds	λ_{abs} (nm)	λ_{em} (nm)	Stokes' shift (nm)	ε ($M^{-1}cm^{-1}$)	QY (φ)	Brightness (B = ε x φ)	Viscosity sensitivity (x)	R^2
**Q-OB**	460	580	120	32046	0.28	8972.88	0.03	0.54
ThT	424	494	70	30000	0.18	5400	0.73	0.99

Source data are provided as a Source Data file.

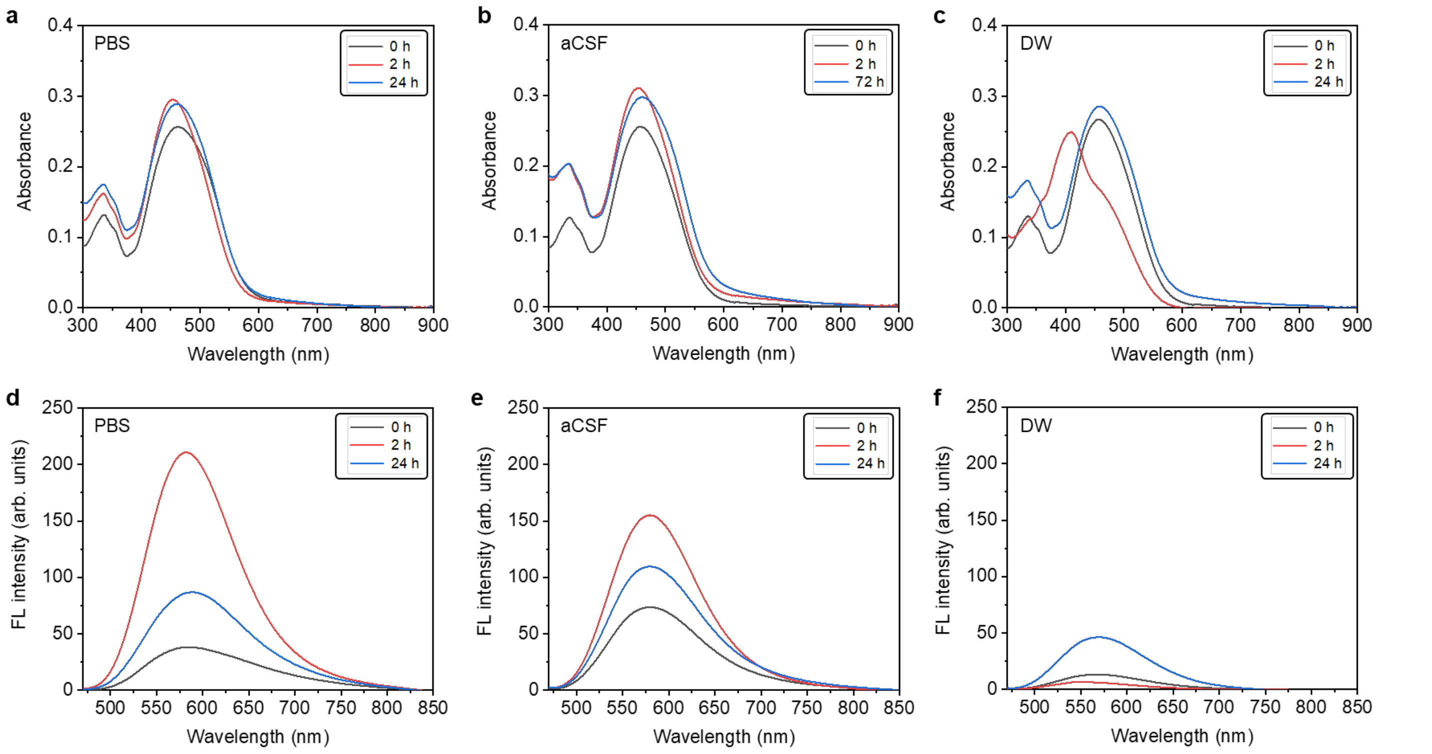

Supplementary Figure 17. Absorbance and fluorescence spectroscopy of Q-OB. Absorption spectra of Q-OB (5 μM) in the presence of $A\beta_{1-42}$ (10 μM) at various time points (0, 2, and 24 h) of incubation in (**a**) PBS (20 mM, pH = 7.4), (**b**) aCSF, and (**c**) deionized water (DW). Fluorescence spectra for identifying Aβ-species (10 μM) using Q-OB (1 μM) at various time points (0, 2, and 24 h) of incubation in (**d**) PBS, (**e**) aCSF, and (**f**) DW (slit 2.5/2.5). Source data are provided as a Source Data file.

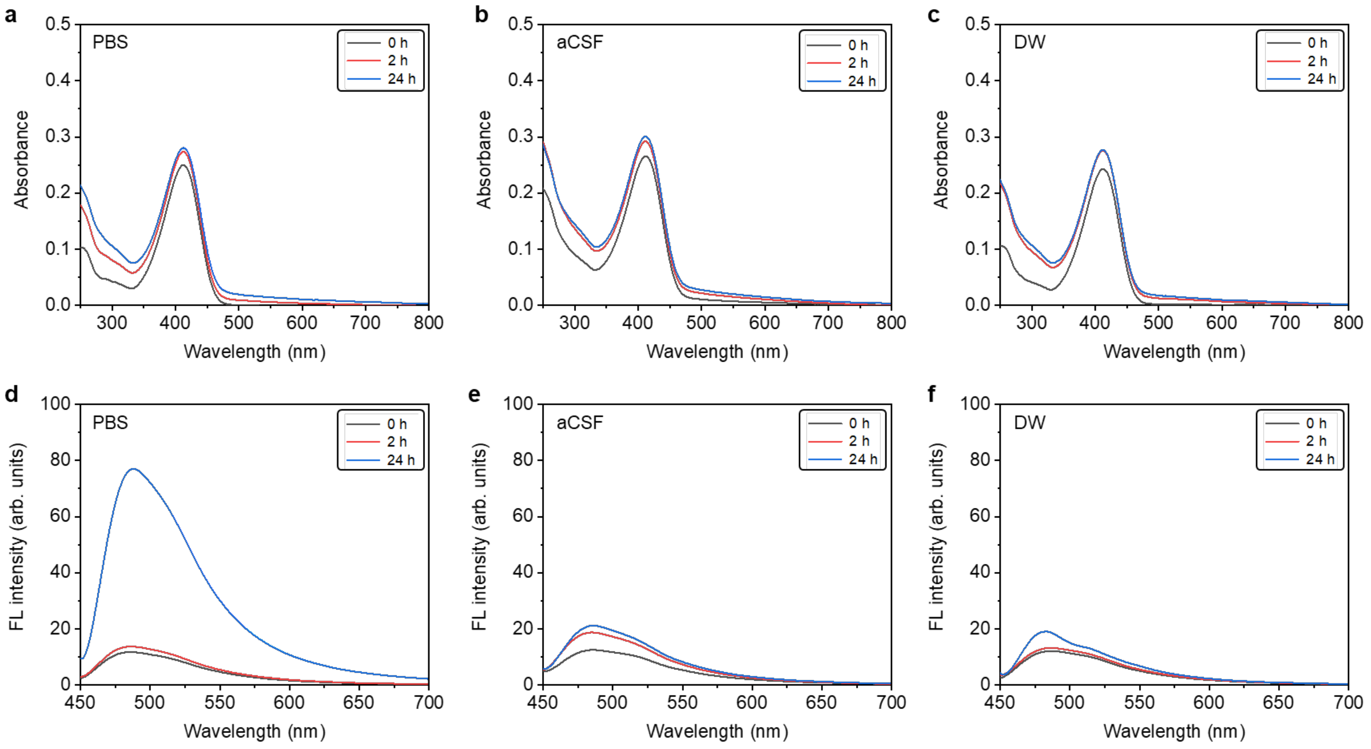

Supplementary Figure 18. Absorbance and fluorescence spectroscopy of ThT. Absorption spectra of ThT (10 μM) in the presence of Aβ_{1-42} (10 μM) at various time points (0, 2, and 24 h) of incubation in (**a**) PBS (20 mM, pH = 7.4), (**b**) aCSF, and (**c**) DW. Fluorescence spectra for identifying Aβ-species (10 μM) using ThT (10 μM) at various time points (0, 2, and 24 h) of incubation in (**d**) PBS, (**e**) aCSF, and (**f**) DW (slit 2.5/2.5). Source data are provided as a Source Data file.

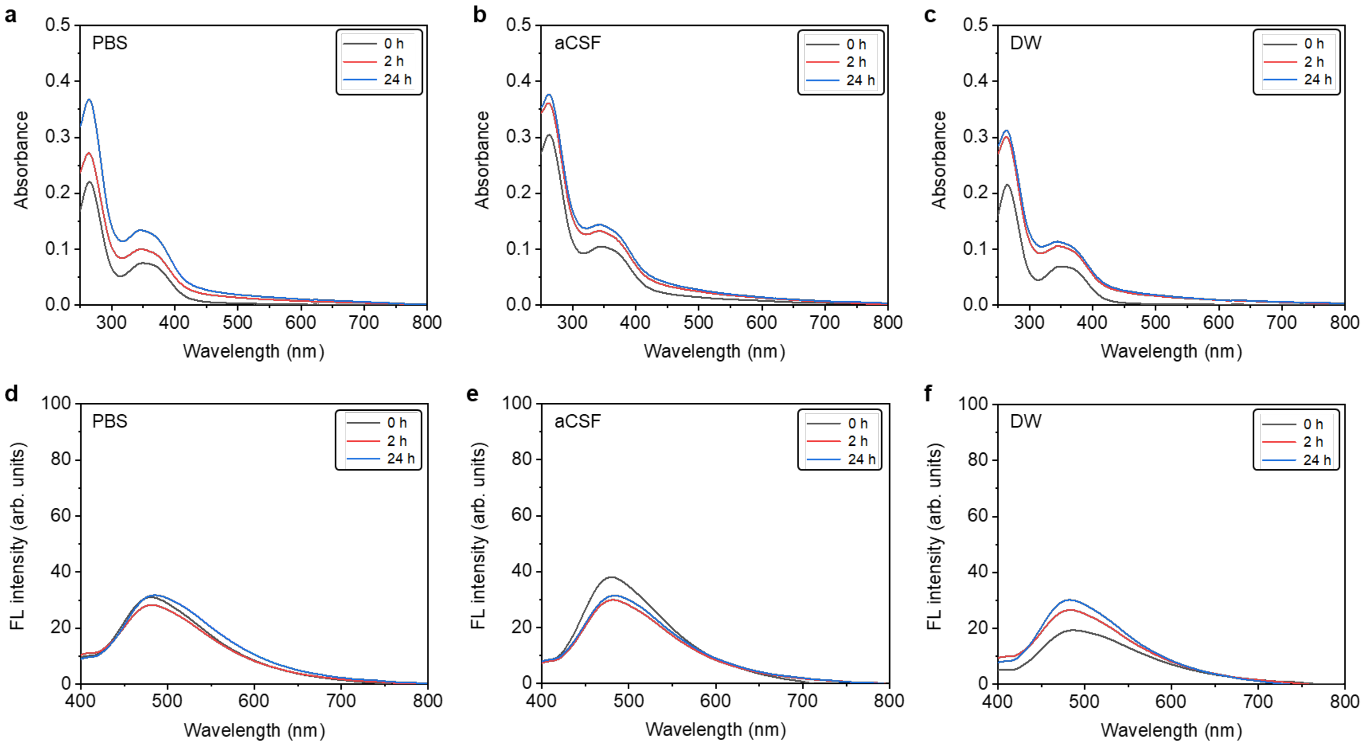

Supplementary Figure 19. Absorbance and fluorescence spectroscopy of 8-anilinonaphthalene-1-sulfonic acid (ANS). Absorption spectra of ANS (10 μM) in the presence of Aβ_{1-42} (10 μM) at various time points (0, 2, and 24 h) of incubation in (**a**) PBS (20 mM, pH = 7.4), (**b**) aCSF, and (**c**) DW. Fluorescence spectra for identifying Aβ_{1-42}-species (10 μM) using ANS (10 μM) at various time points (0, 2, and 24 h) of incubation in (**d**) PBS, (**e**) aCSF, and (**f**) DW (slit 2.5/2.5). Source data are provided as a Source Data file.

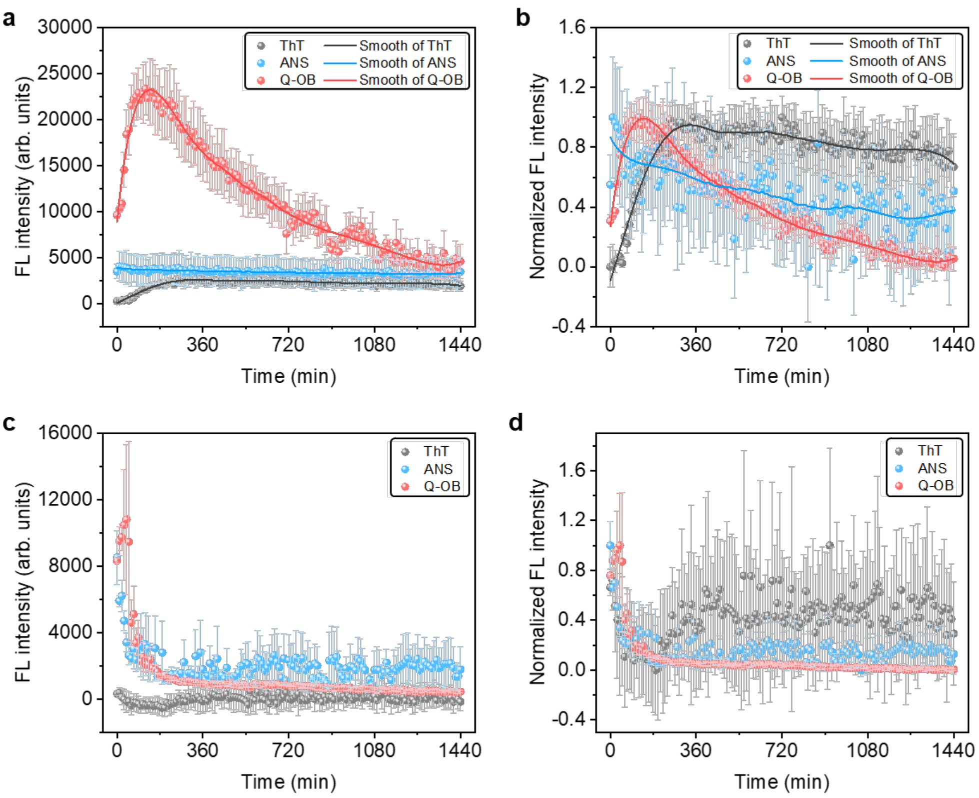

Supplementary Figure 20. a Identification of A$\beta_{1\text{–}42}$ aggregation kinetics using Q-OB (1 µM), ThT (10 µM), and ANS (10 µM) under the coincubation at 37°C for 24 h with A$\beta_{1\text{–}42}$ (10 µM) in PBS (20 mM, pH = 7.4) and (**b**) normalized plot obtained from (**a**). A$\beta_{1\text{–}42}$ aggregation kinetics using Q-OB, ThT, and ANS under the coincubation at 37°C for 24 h with A$\beta_{1\text{–}42}$ monomer (10 µM) in (**c**) aCSF and (**d**) DW. Error ranges represent s.d. (n = 4 independent experiments). Source data are provided as a Source Data file.

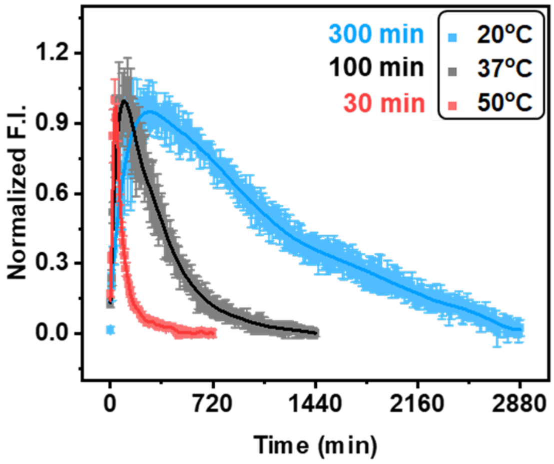

Supplementary Figure 21. Identification of A$\beta_{1\text{–}42}$ aggregation kinetics by Q-OB under coincubation ($\lambda_{ex}/\lambda_{em}$ = 460/580 nm) at 20, 37, and 50°C (full-time range of Figure 2h in main text). Error ranges represent s.d. (n = 3 independent experiments). Source data are provided as a Source Data file.

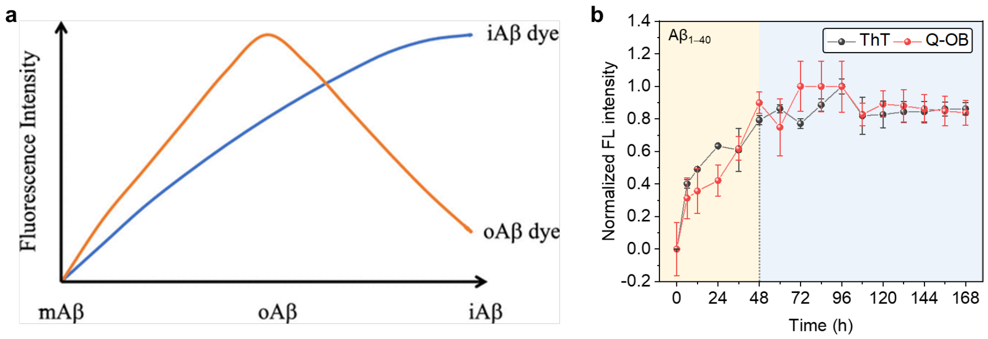

Supplementary Figure 22. a Hypothetical pattern of fluorescence intensities of Aβ-specific fluorophores in kinetic assay reported in our previous work[22]. The fluorescence intensity of aqueous-insoluble Aβ fibrils and Aβ oligomers fluorophores during Aβ self-aggregation with fibrillar Aβ concentration increasing from Aβ monomers, while Aβ oligomer levels increase then disappearing as they aggregate into matured fibrils. **b** Time-dependent plot indicated the change in the fluorescence intensity of Q-OB and ThT in the presence of $A\beta_{1-40}$ for 168 h. Data are represented as mean ± s.d. with n = 3 independent experiments. The abbreviated words are mAβ, amyloid-β monomers; oAβ, amyloid-β oligomers; iAβ, insoluble amyloid-β fibrils. Source data underlying **b** are provided as a Source Data file.

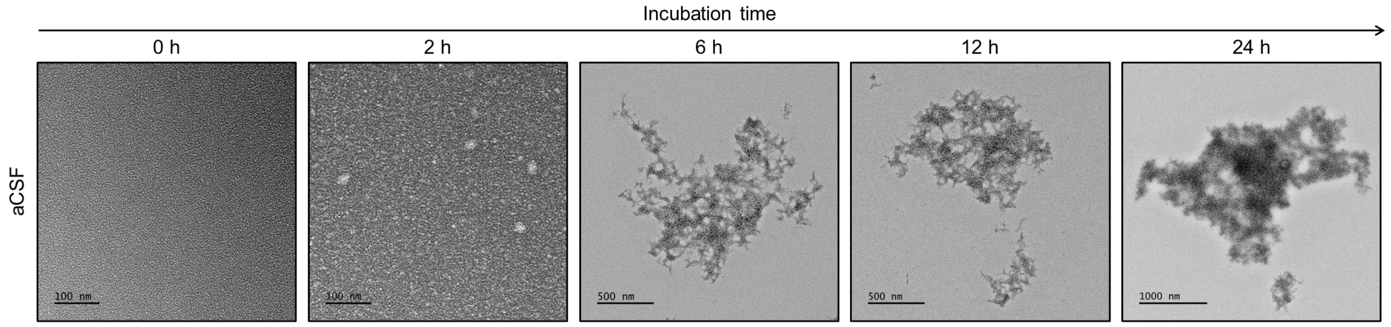

Supplementary Figure 23. Biophysical characterization using transmission electron microscopy (TEM) images of $A\beta_{1-42}$ at specific time points (0, 2, 6, 12, and 24 h) of fibrillogenesis in aCSF.

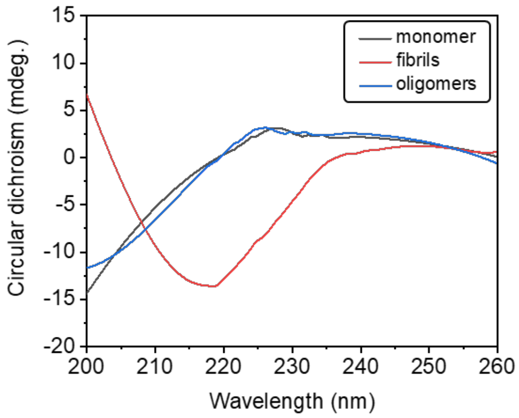

Supplementary Figure 24. Biophysical characterization of $A\beta_{1-42}$ species, including monomer, oligomers, and fibrils, by circular dichroism spectra. Source data are provided as a Source Data file.

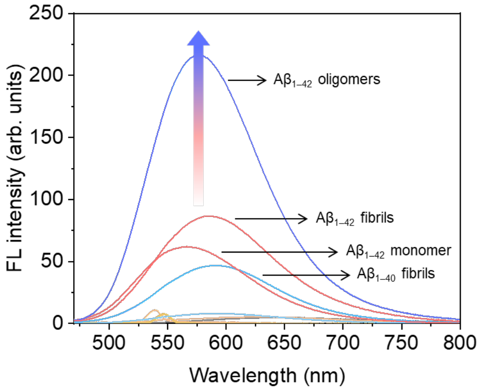

Supplementary Figure 25. Fluorescent spectra result of Figure 3b in main text. Fluorescence spectra of various Aβ-species (10 μM) and Q-OB (5 μM) with Aβ-species (10 μM). Source data are provided as a Source Data file.

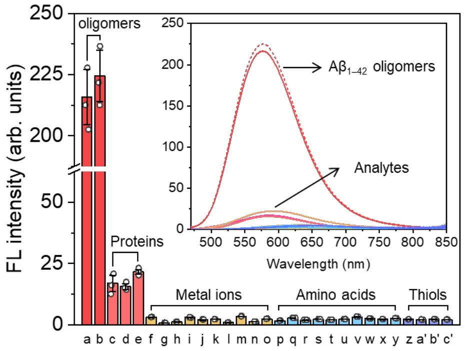

Supplementary Figure 26. Fluorescence intensity of Q-OB (5 μM) with various potential interferents, a: $Aβ_{1–42}$ oligomers (10 μM); b: $Aβ_{1–42}$ oligomers (10 μM) mixed with all analytes; c–e: proteins (10 μM, c: human serum albumin (HSA); d: bovine serum albumin (BSA); e: phosphorylated tau (p-Tau)); f–o: metal ions (1 mM, f: Mg^{2+}; g: Fe^{3+}; h: Fe^{2+}; i: Zn^{2+}; j: Ni^{2+}; k: K^{+}; l: Al^{3+}; m: Ca^{2+}; n: Cu^{2+}; o: Na^{+}); p–y: amino acids (1 mM, p: Glu; q: Phe; r: Tyr; s: Gly; t: Trp; u: Lys; v: Asn; w: His; x: Met; y: Val); z–c': thiols (1 mM, z: GSH; a': Hcy; b': DTT; c': Cys). Inset: Fluorescence spectra of Q-OB with various potential interferents. Error ranges represent s.d. (n = 3 independent experiments), slit 2.5/2.5. Source data are provided as a Source Data file.

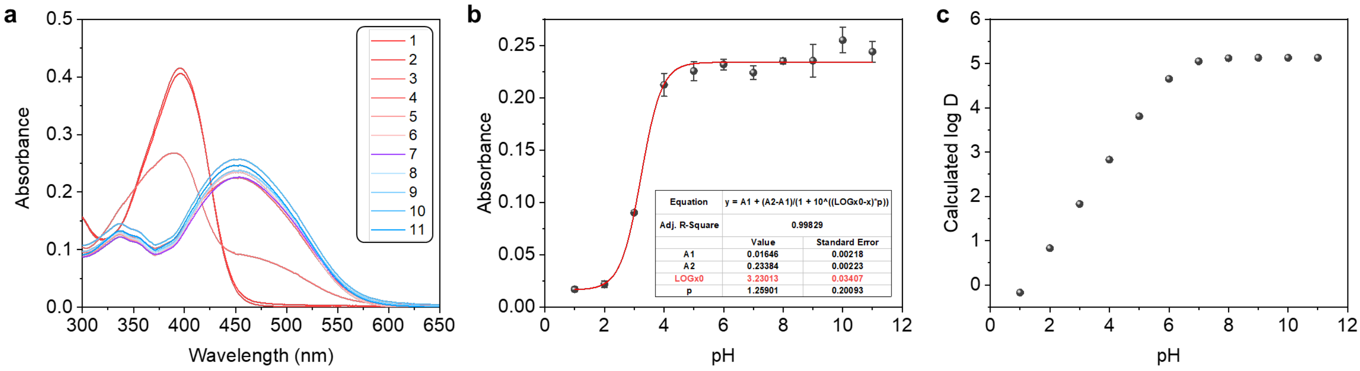

Supplementary Figure 27. pH-dependent absorbance changes. **a** Absorbance spectra of Q-OB at various pH levels (pH = 1–11). **b** The pH-dependent absorbance at 460 nm, with non-linear pK_a fitting. **c** pH-dependent calculated log D values. log D was calculated using the general equation for bases: $log\ D = log\ P - log[1 + 10^{(pK_a - pH)}]$, with calculated p$K_a$ = 6.3 from Molinspiration cheminformatics applet (https://www.molinspiration.com/) and log P = 5.13. Error bars represent s.d. derived from n = 3 independent experiments. Source data are provided as a Source Data file.

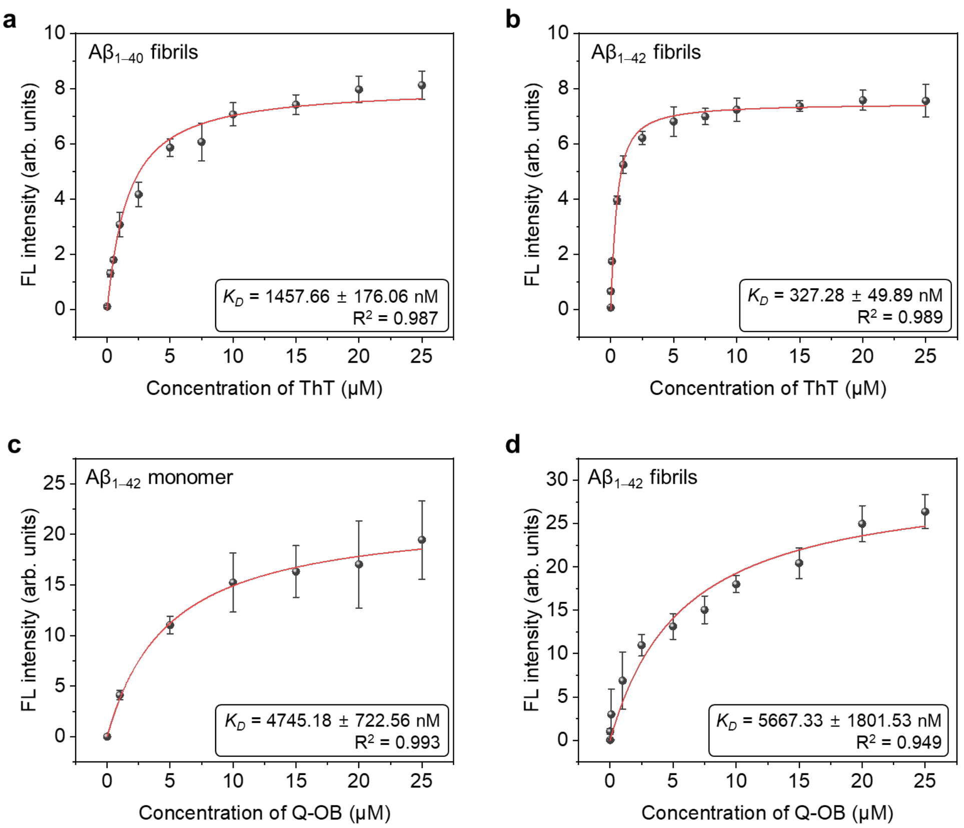

Supplementary Figure 28. Saturation binding curved of (**a**) A$\beta_{1\text{–}40}$ fibrils and (**b**) A$\beta_{1\text{–}42}$ fibrils as a function of ThT (0–25 µM) as a reference. Saturation binding curved of (**c**) A$\beta_{1\text{–}42}$ monomer and (**d**) A$\beta_{1\text{–}42}$ fibrils as a function of Q-OB (0–25 µM). Error ranges represent s.d. (*n* = 3 independent experiments), slit 2.5/2.5. Source data are provided as a Source Data file.

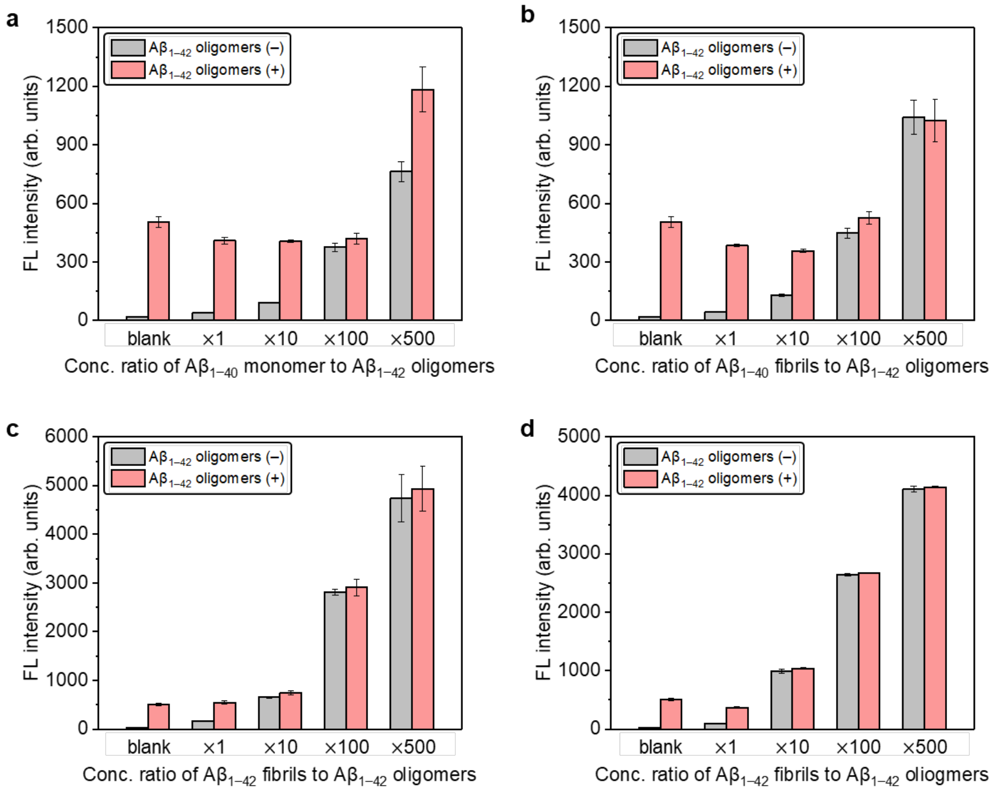

Supplementary Figure 29. Identification of cross-reactivity of Q-OB for Aβ-species. The fluorescence emission intensity of Q-OB (1 μM) with $Aβ_{1–42}$ oligomers (0.1 μM) in PBS (10 mM, pH = 7.4) containing 1% DMSO in comparison with varying concentrations of cross-reactive interferent, including (**a**) $Aβ_{1–40}$ monomer, (**b**) $Aβ_{1–40}$ fibrils, (**c**) $Aβ_{1–42}$ monomer, and (**d**) $Aβ_{1–42}$ fibrils. Error ranges represent s.d. (*n* = 3 independent experiments), slit 2.5/2.5. Source data are provided as a Source Data file.

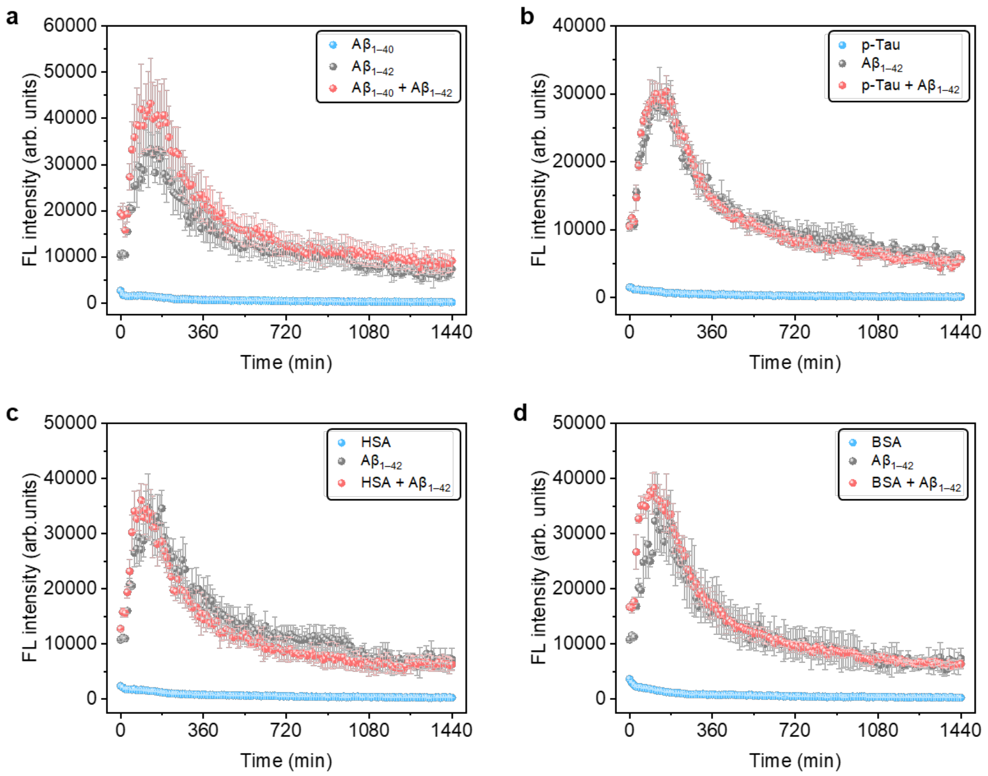

Supplementary Figure 30. Identification of Aβ aggregation kinetics using Q-OB (1 μM) in the presence of $A\beta_{1\text{–}42}$ (10 μM) in PBS (20 mM, pH = 7.4) in comparison with potential interferents including (**a**) $A\beta_{1\text{–}40}$ (10 μM), (**b**) phosphorylated tau (p-Tau) (10 μM), (c) human serum albumin (HSA) (40 mg/mL), and (**d**) bovine serum albumin (BSA) (40 mg/mL). Error ranges represent s.d. (n = 3 independent experiments). Source data are provided as a Source Data file.

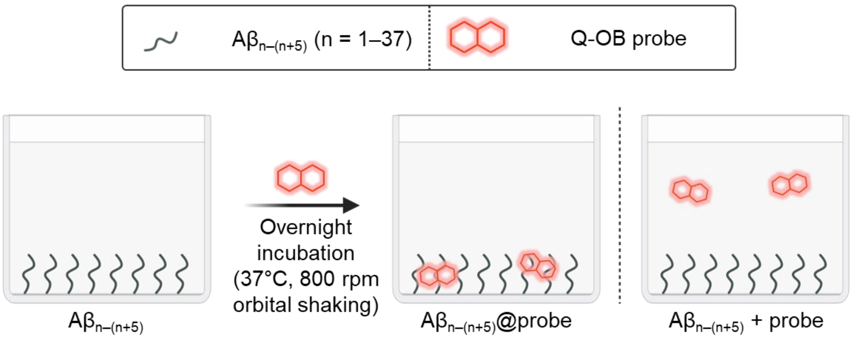

Supplementary Figure 31. Identification of binding residues of Q-OB. Experimental scheme for identification of Q-OB binding site for $A\beta_{n\text{–}(n+5)}$ fragments of $A\beta_{1\text{–}42}$[24]. Created with BioRender.com.

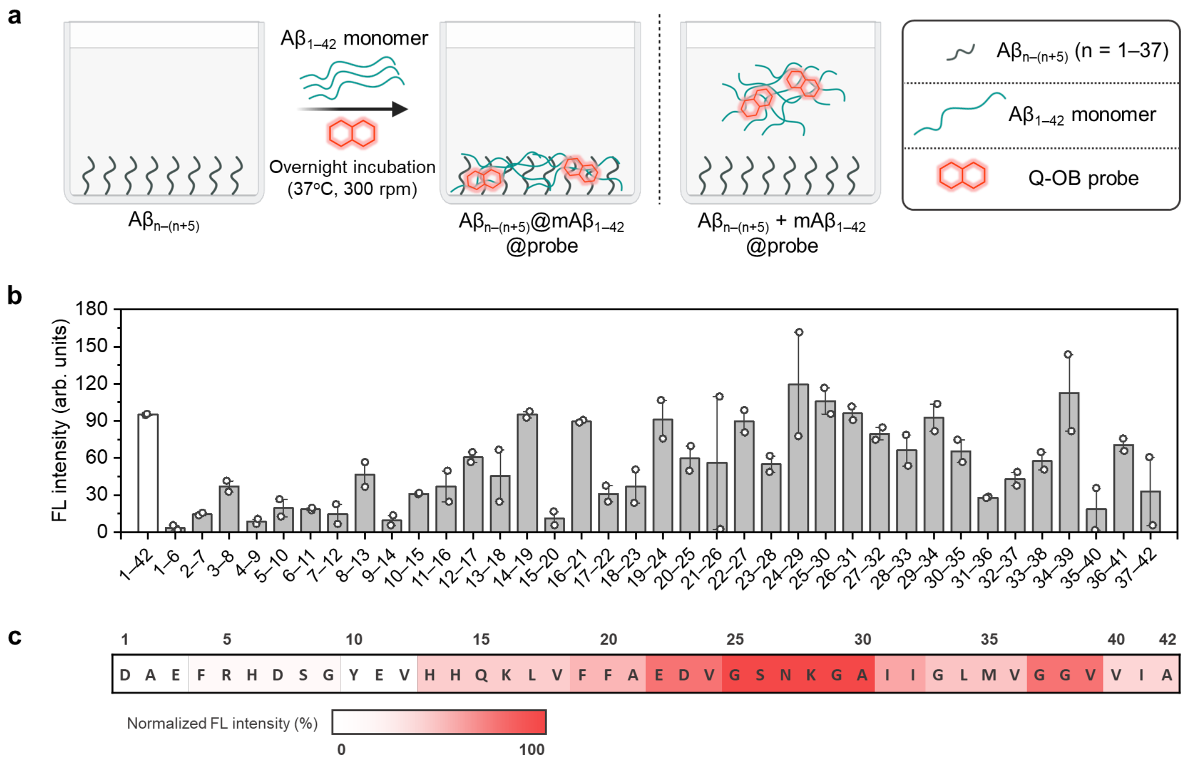

Supplementary Figure 32. Identification of binding residues of Q-OB. **a** Experimental scheme for identification of Q-OB binding site for A$\beta_{n-(n+5)}$ fragments of Aβ_{1-42}, with additional full-length Aβ_{1-42}[24]. **b** The data of fluorescent values of Q-OB and (**c**) the heatmap displays the spectrum of binding intensity on A$\beta_{n-(n+5)}$ sequence (white to red, 0–100%) ($\lambda_{ex}/\lambda_{em}$ = 500/620 nm). Error ranges represent s.d. (n = 2 independent experiments). Panel **a** was created with BioRender.com. Source data underlying **b** are provided as a Source Data file.

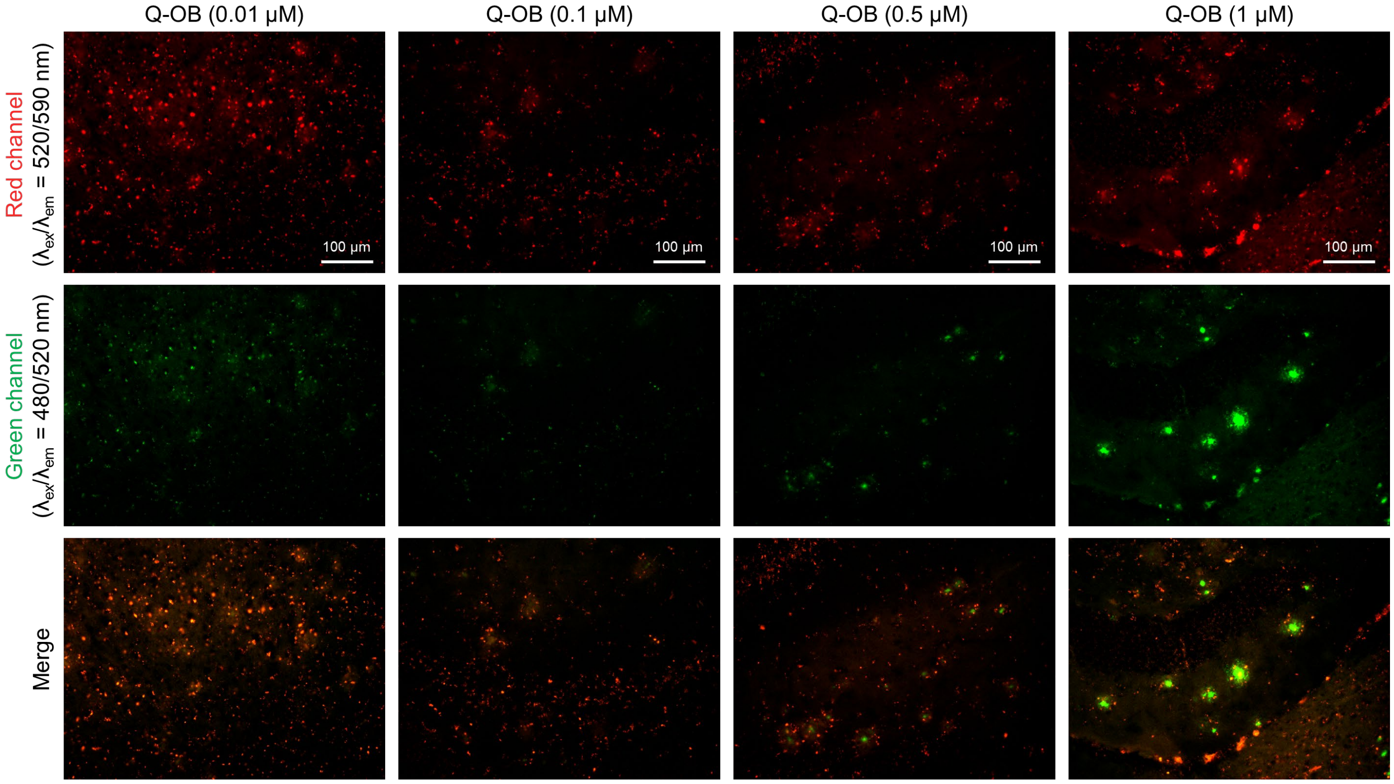

Supplementary Figure 33. Fluorescence images of cortex area of brain tissue slice from 12-month-old 5xFAD transgenic Alzheimer's disease (AD) model mice stained by various concentrations of Q-OB (0.01, 0.1, 0.5, and 1 μM) (red channel; $\lambda_{em}/\lambda_{ex}$ = 520/590 nm and green channel; $\lambda_{em}/\lambda_{ex}$ = 480/520 nm). Scale bars indicate the length stated in the figures (scale bar: 100 μm).

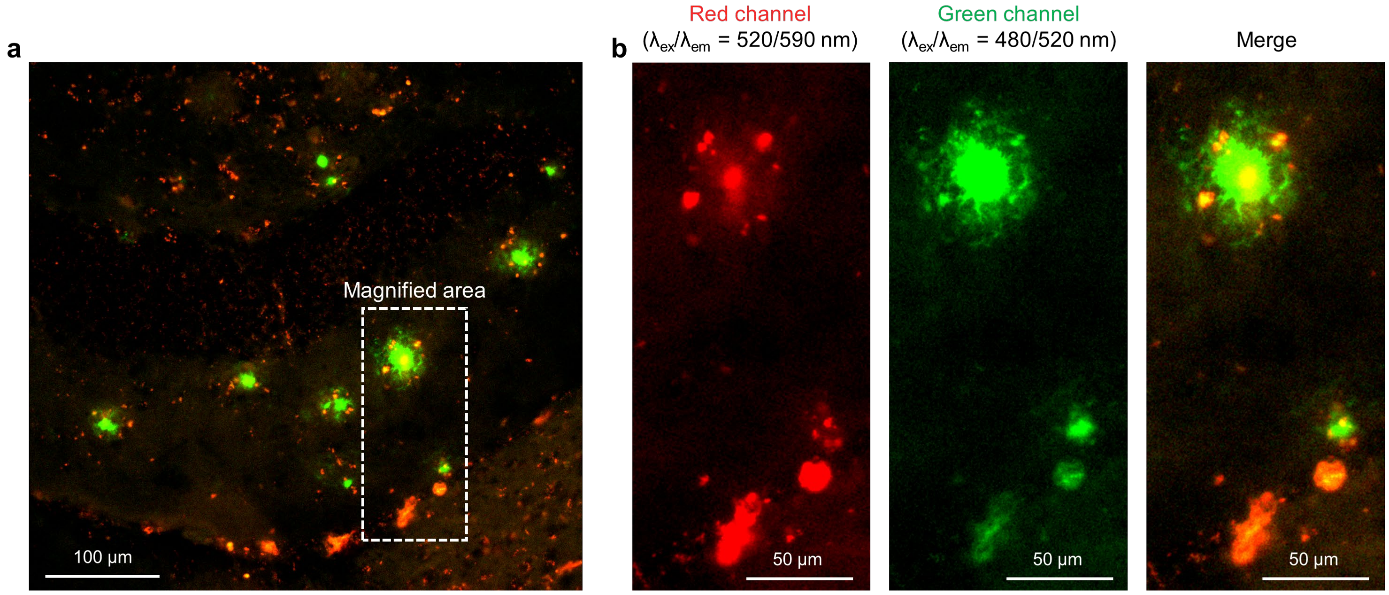

Supplementary Figure 34. a Fluorescence images of cortex area of the brain tissue slice of 12-month-old 5xFAD transgenic AD model mouse stained by Q-OB (1 μM) (red channel; $\lambda_{em}/\lambda_{ex}$ = 520/590 nm and green channel; $\lambda_{em}/\lambda_{ex}$ = 480/520 nm). **b** Magnified images of Aβ aggregates at the white dot-line boxed area. Scale bars indicate the length stated in the figures. Scale bar: 100 μm (**a**), 50 μm (**b**).

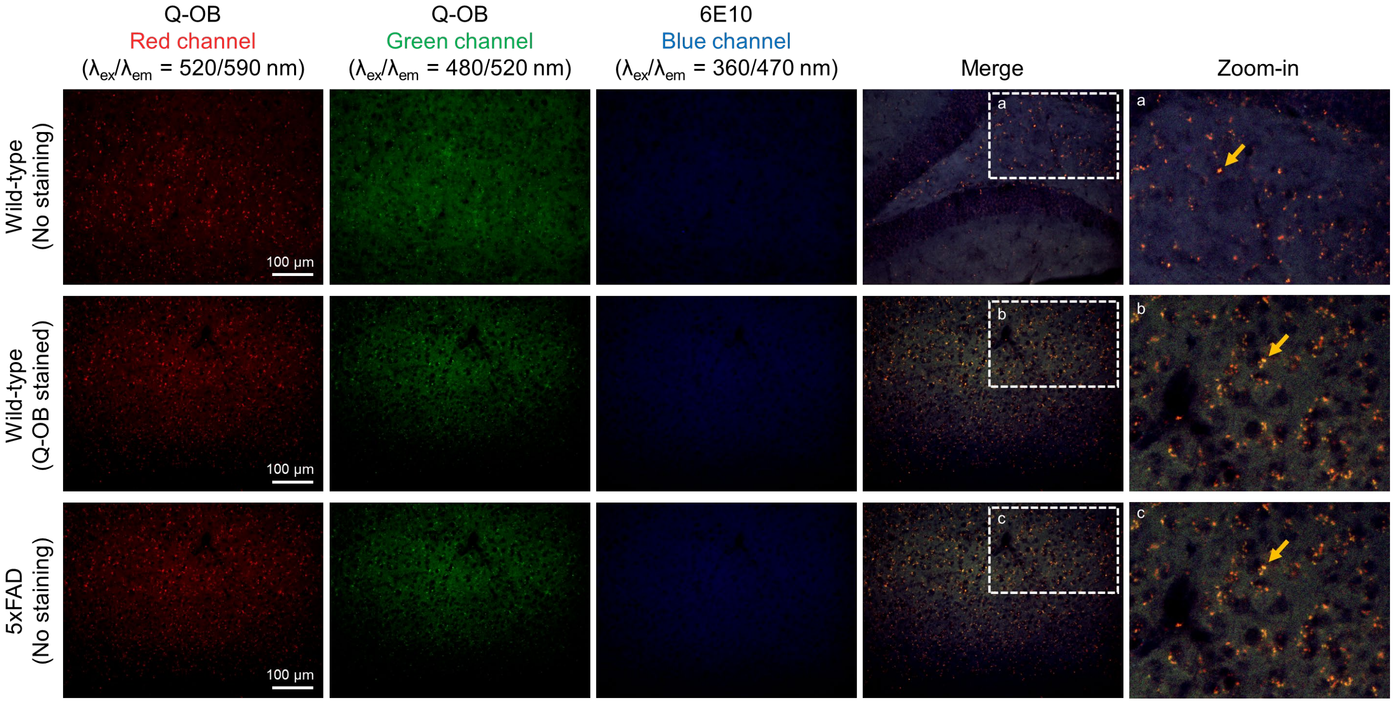

Supplementary Figure 35. Fluorescence images of cortex area of the brain tissue isolated from 6-month-old B6 wild-type mouse and 16.5-month-old 5xFAD transgenic AD model mouse with or without staining using Q-OB (1 μM, red and green channels) and 6E10 (anti-Aβ antibody, 1:200, blue channel) (red channel; $\lambda_{em}/\lambda_{ex}$ = 520/590 nm, green channel; $\lambda_{em}/\lambda_{ex}$ = 480/520 nm, and blue channel; $\lambda_{em}/\lambda_{ex}$ = 360/470 nm). White dot-line boxes indicate magnified area for a, b, and c, respectively, and yellow arrows in zoom-in images demonstrate autofluorescence. Scale bars indicate the length stated in the figures (scale bar: 100 μm).

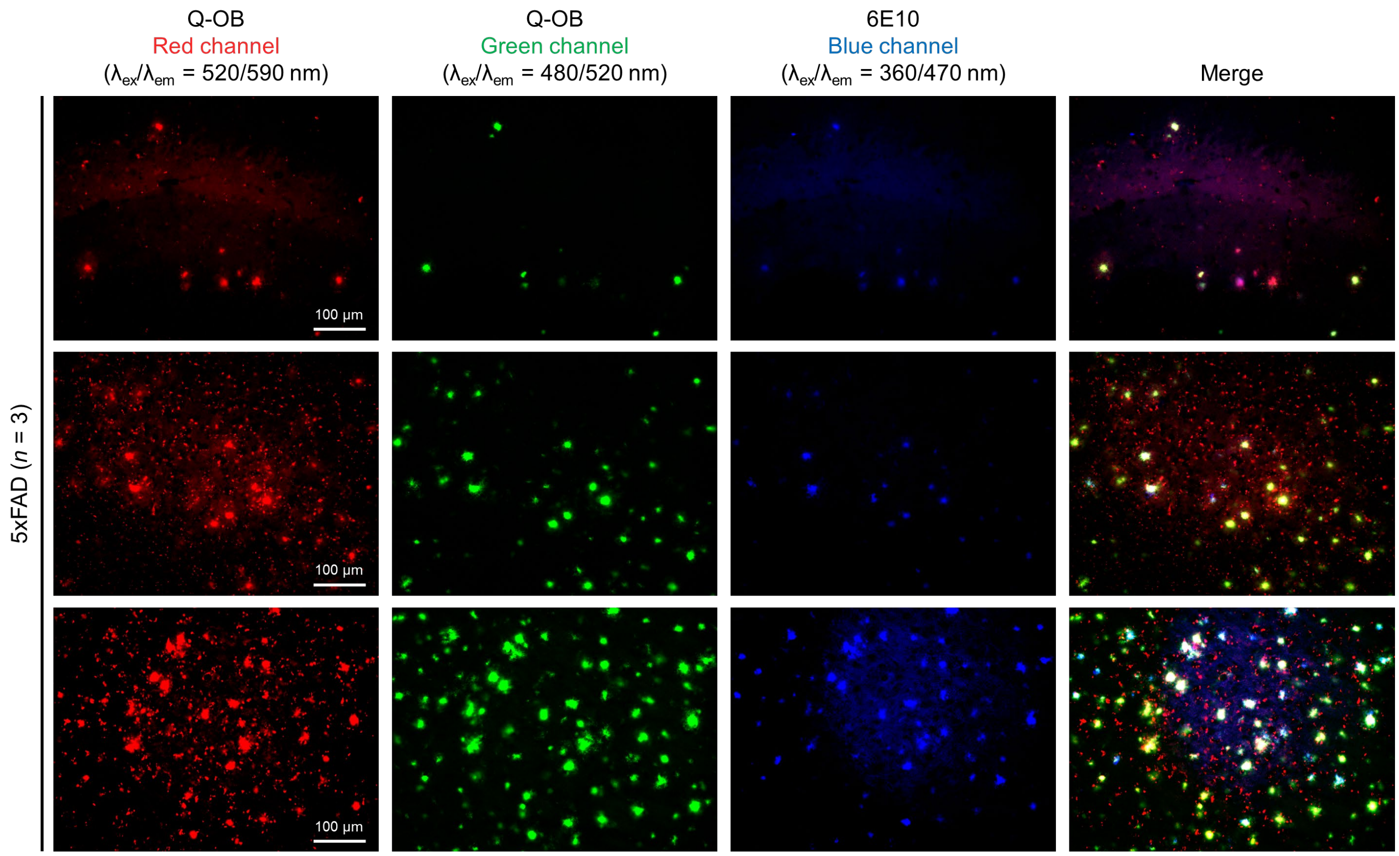

Supplementary Figure 36. Fluorescence images of cortex area of the brain tissue slice (16-month-old 5xFAD transgenic AD model mouse) stained by Q-OB (1 μM, red and green channels) and 6E10 (anti-Aβ antibody, 1:200, blue channel) (red channel; $\lambda_{em}/\lambda_{ex}$ = 520/590 nm, green channel; $\lambda_{em}/\lambda_{ex}$ = 480/520 nm, and blue channel; $\lambda_{em}/\lambda_{ex}$ = 360/470 nm) (*n* = 3 biologically independent samples). Scale bars indicate the length stated in the figure (scale bar: 100 μm).

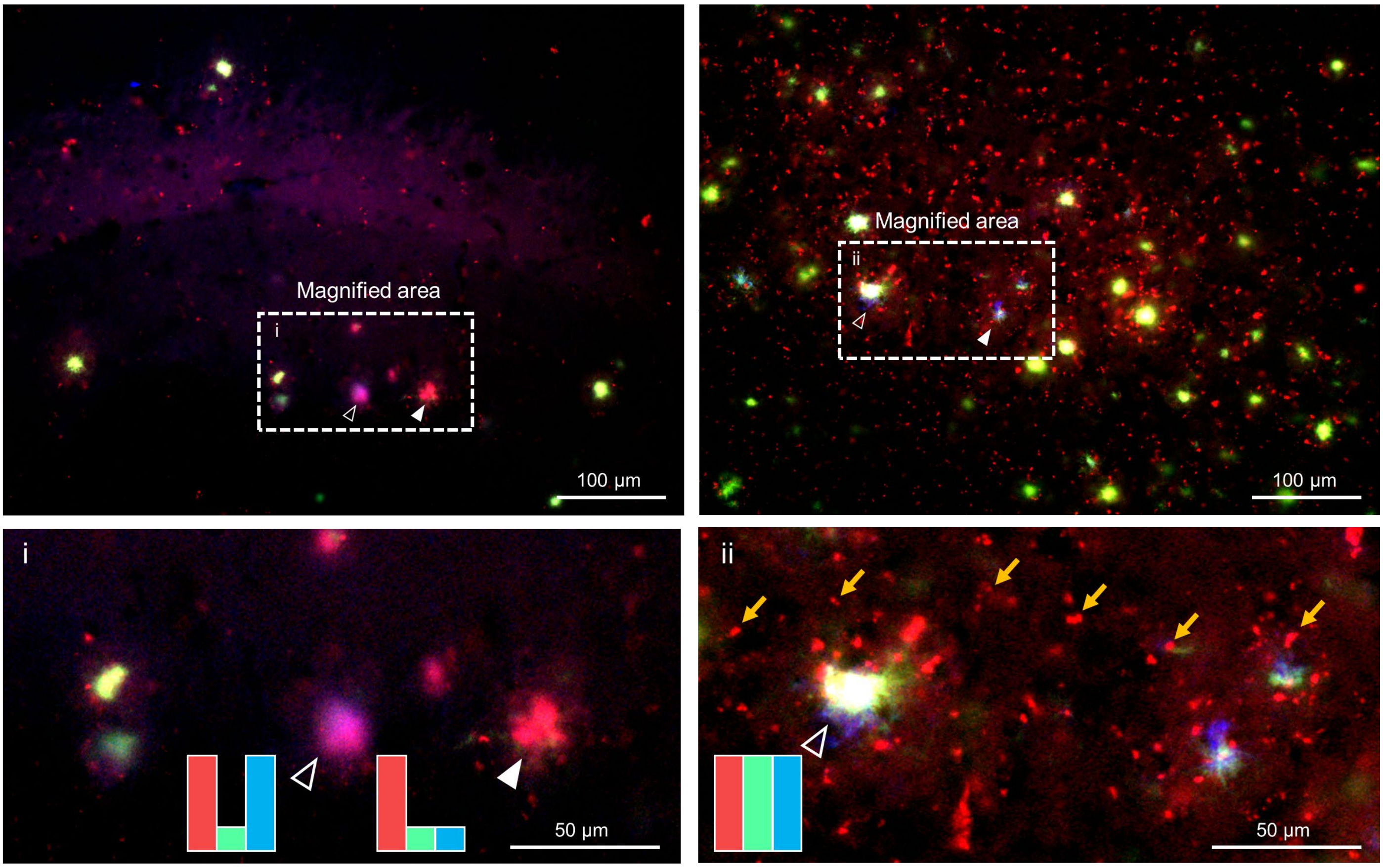

Supplementary Figure 37. Fluorescence images of cortex area of the brain tissue slice from 16-month-old 5xFAD transgenic AD model mouse stained by Q-OB (1 μM, red and green channel) and 6E10 (anti-Aβ antibody, 1:200, blue channel) (red channel; $\lambda_{em}/\lambda_{ex}$ = 520/590 nm, green channel; $\lambda_{em}/\lambda_{ex}$ = 480/520 nm, and blue channel; $\lambda_{em}/\lambda_{ex}$ = 360/470 nm). Magnified images of Aβ aggregates at the white dot-line boxed area for (i) and (ii), respectively. i) Stained Aβ aggregates are indicated by hollowed arrow (overlap of red and blue channels), and white arrow (red channel without overlapping). ii) Stained Aβ aggregates are marked by hollowed arrow (overlapping red, green, and blue channels). Yellow arrows in zoom-in images demonstrate autofluorescence. Scale bars indicate the length stated in the figures. Scale bar: 100 μm (upper panels); 50 μm (bottom panels).

Supplementary Table 4. Information of postmortem cerebral hippocampal tissue specimens.

No	Diagnosis	Age	Sex	PMI (h)	APOE	Staining methods
1	ADD	54.2	M	49.5	ε3/ε3	Q-OB/6E10 co-staining
2	ADD	70.3	M	13.5	ε3/ε3	
3	ADD	73.3	F	7.5	ε3/ε4	
4	ADD	80.3	F	47	ε2/ε3	
5	ADD	80.6	M	24	ε3/ε3	
6	CN	72.6	M	42.5	ε3/ε3	
7	CN	73.5	M	22	ε3/ε3	
8	CN	78.3	M	46	ε3/ε3	
9	CN	78.8	F	19	ε2/ε3	
10	CN	79.3	M	57	ε3/ε3	
11	ADD	63.5	M	43	ε2/ε3	Q-OB/4G8 co-staining
12	ADD	83	F	34	ε3/ε3	
13	CN	59	F	30	ε3/ε3	

Written informed consent was obtained from each participant or their legal guardian. Diagnostic information was designated by the Victorian Brain Bank Network (VBBN), supported by The Florey, The Alfred, and the Victorian Institute of Forensic Medicine. The abbreviated words are Alzheimer's disease dementia (ADD), Cognitive normal (CN), Female (F), Male (M), post-mortem interval (PMI), and apolipoprotein E (APOE).

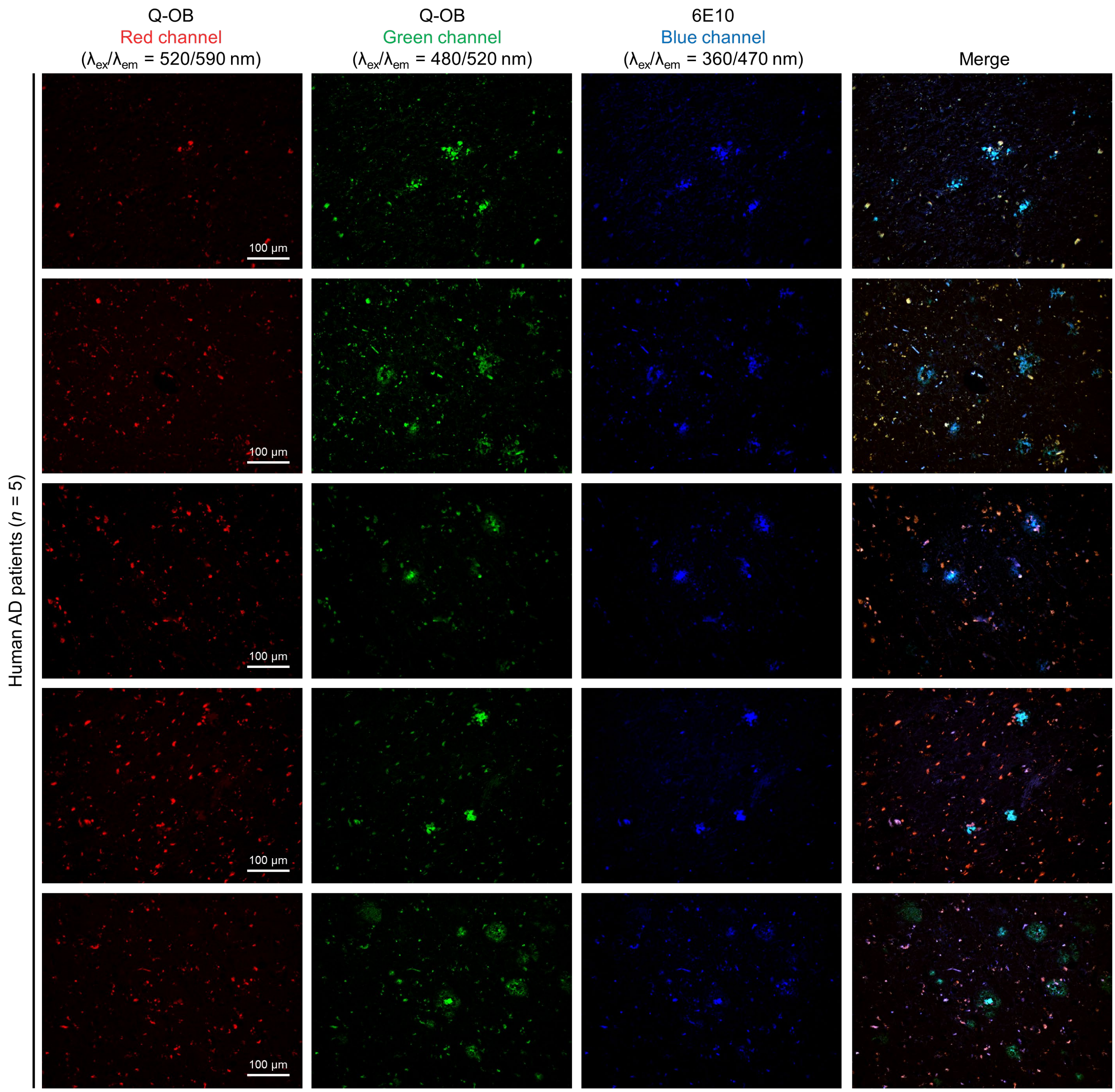

Supplementary Figure 38. Fluorescence images of hippocampus area of the brain tissues (AD-diagnosed patients) stained by Q-OB (1 µM, red and green channels) and 6E10 (anti-Aβ antibody, 1:200, blue channel) (red channel; $\lambda_{em}/\lambda_{ex}$ = 520/590 nm, green channel; $\lambda_{em}/\lambda_{ex}$ = 480/520 nm, and blue channel; $\lambda_{em}/\lambda_{ex}$ = 360/470 nm) (*n* = 5 individually independent samples). Scale bars indicate the length stated in the figures (scale bar: 100 µm).

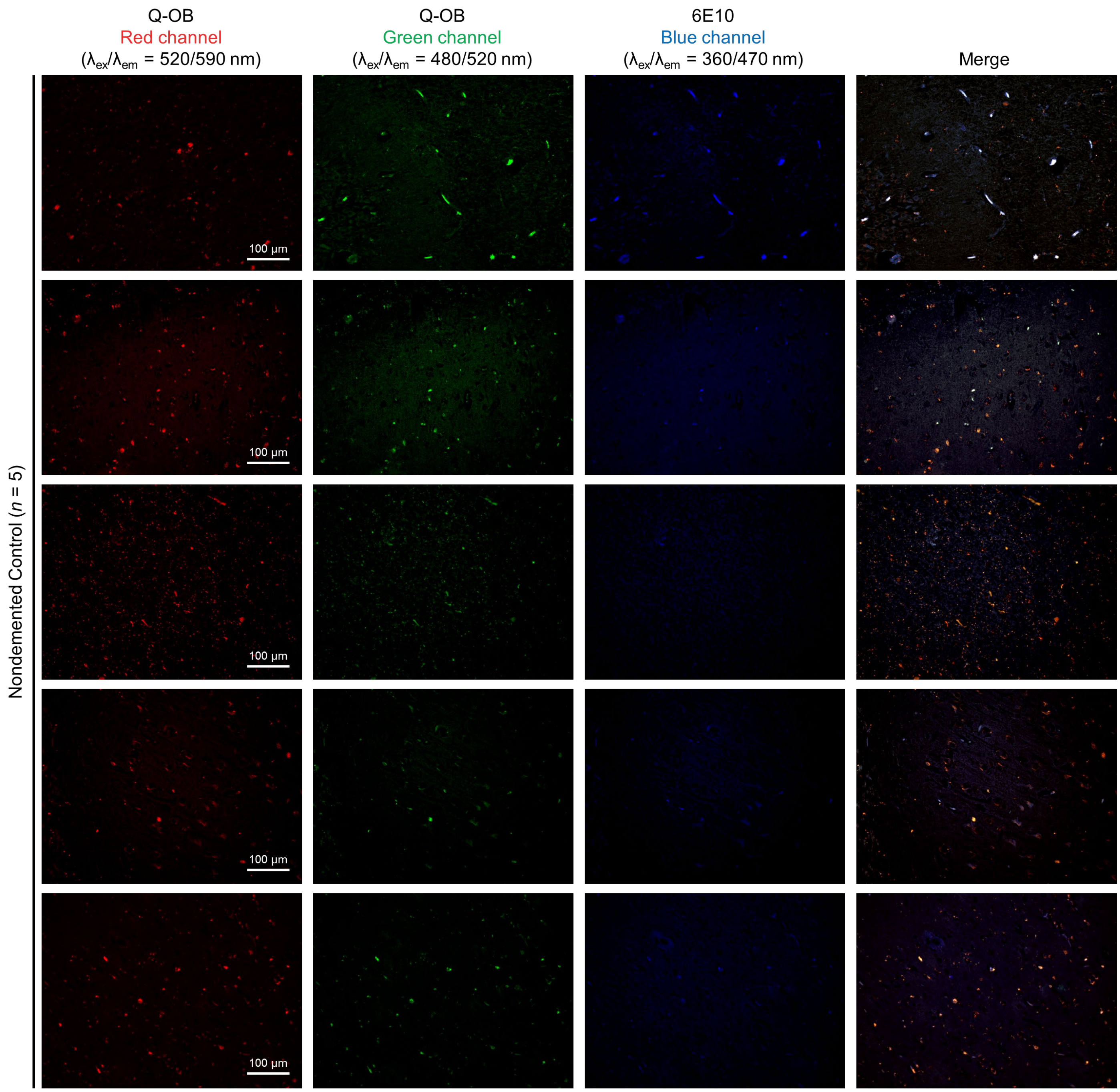

Supplementary Figure 39. Fluorescence images of hippocampus area of the brain tissues of healthy human (non-AD-patient) controls stained by Q-OB (1 μM, red and green channels) and 6E10 (anti-Aβ antibody, 1:200, blue channel) (red channel; $\lambda_{em}/\lambda_{ex}$ = 520/590 nm, green channel; $\lambda_{em}/\lambda_{ex}$ = 480/520 nm, and blue channel; $\lambda_{em}/\lambda_{ex}$ = 360/470 nm) (*n* = 5 individually independent samples). Scale bars indicate the length stated in the figures (scale bar: 100 μm).

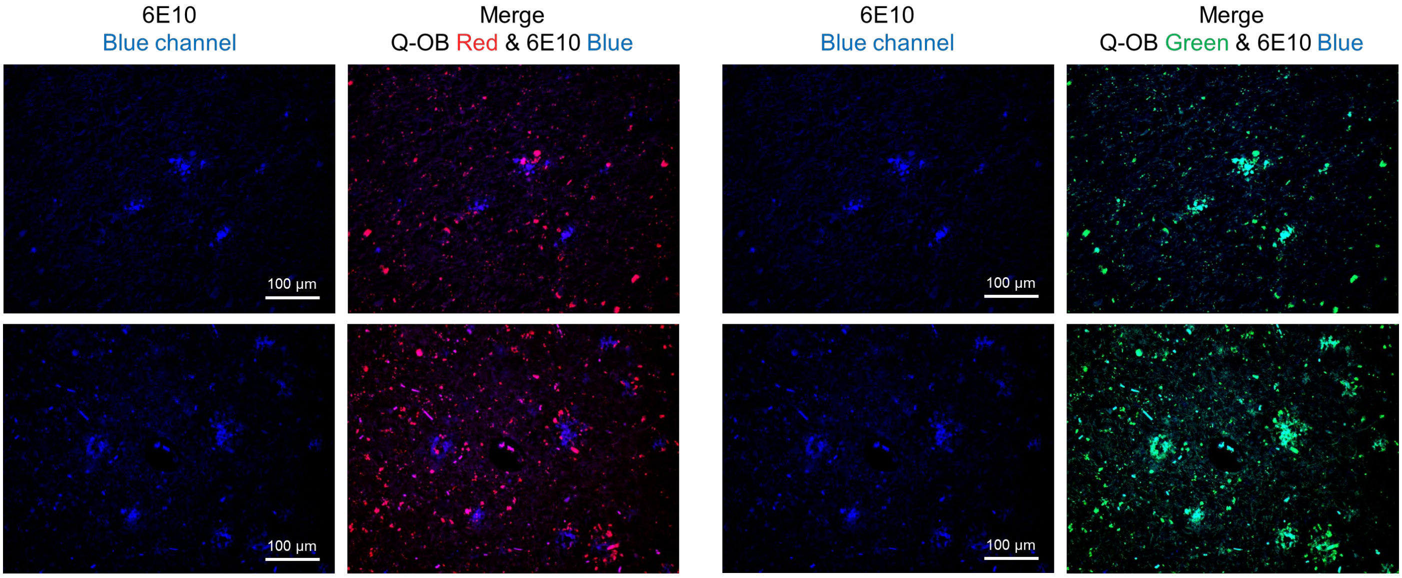

Supplementary Figure 40. Fluorescence images of hippocampus area of the brain tissues from human AD patients stained by Q-OB (1 μM, red and green channels) and 6E10 (anti-Aβ antibody, 1:200, blue channel) (red channel; $\lambda_{em}/\lambda_{ex}$ = 520/590 nm, green channel; $\lambda_{em}/\lambda_{ex}$ = 480/520 nm, and blue channel; $\lambda_{em}/\lambda_{ex}$ = 360/470 nm). Scale bars indicate the length stated in the figures (scale bar: 100 μm).

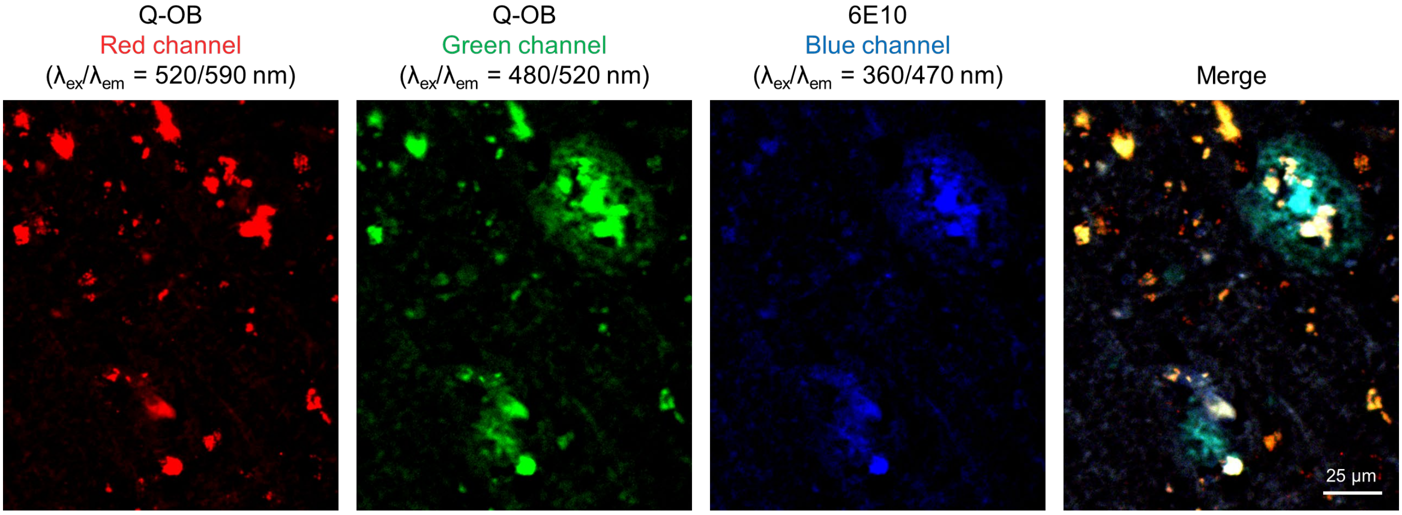

Supplementary Figure 41. Fluorescence images of hippocampus area of the brain tissues collected from human AD patients. a) Magnified images of Aβ aggregates stained by Q-OB (1 μM, red and green channels) and 6E10 (anti-Aβ antibody, 1:200, blue channel) (red channel; $\lambda_{em}/\lambda_{ex}$ = 520/590 nm, green channel; $\lambda_{em}/\lambda_{ex}$ = 480/520 nm, and blue channel; $\lambda_{em}/\lambda_{ex}$ = 360/470 nm). Scale bars indicate the length of stated in the figures (scale bar: 25 μm).

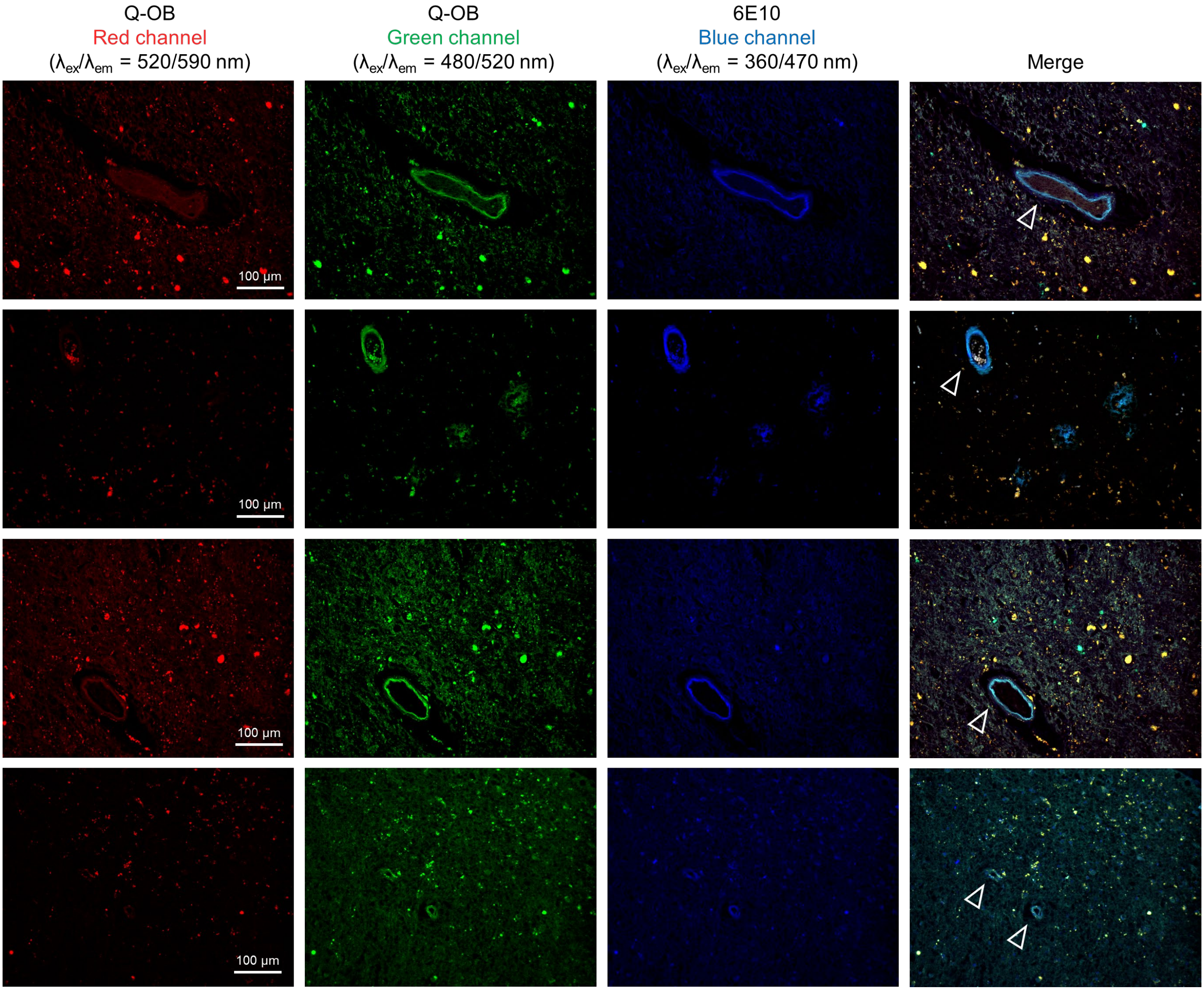

Supplementary Figure 42. Fluorescence images of hippocampus area of the AD patients' brain tissues stained by Q-OB (1 µM, red and green channels) and 6E10 (anti-Aβ antibody, 1:200, blue channel) (red channel; $\lambda_{em}/\lambda_{ex}$ = 520/590 nm, green channel; $\lambda_{em}/\lambda_{ex}$ = 480/520 nm, and blue channel; $\lambda_{em}/\lambda_{ex}$ = 360/470 nm). White-hollowed arrows in merge images demonstrate vascular Aβ deposits. Scale bars indicate the length stated in the figures (scale bar: 100 µm).

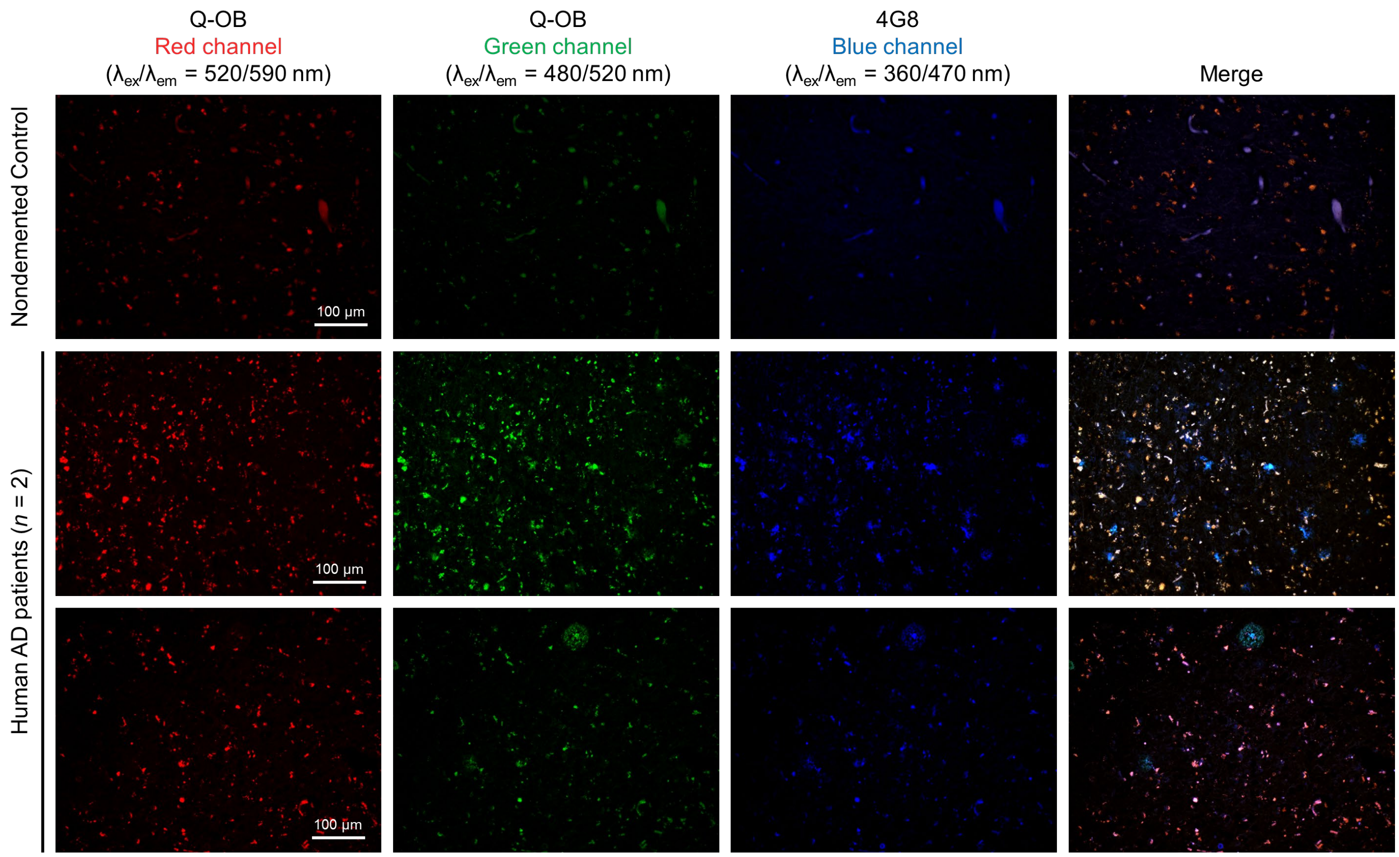

Supplementary Figure 43. Fluorescence images of hippocampus area of the brain tissue of healthy human (non-AD-patient) and human AD patients stained by Q-OB (1 μM, red and green channels) and 4G8 (anti-Aβ antibody, 1:200, blue channel) (red channel; $\lambda_{em}/\lambda_{ex}$ = 520/590 nm, green channel; $\lambda_{em}/\lambda_{ex}$ = 480/520 nm, and blue channel; $\lambda_{em}/\lambda_{ex}$ = 360/470 nm) (*n* = 3 individually independent samples). Scale bars indicate the length stated in the figures (scale bar: 100 μm).

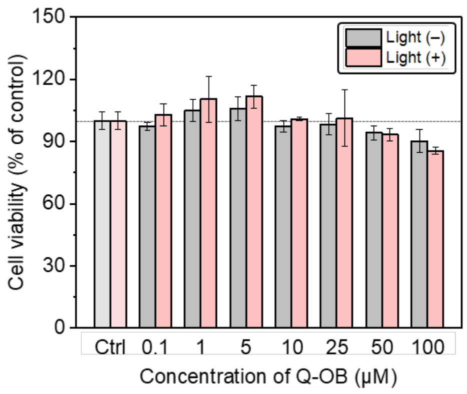

Supplementary Figure 44. Cytotoxicity of Q-OB in vitro. Cell viabilities of SH-SY5Y cell treated with Q-OB (0, 0.1, 1, 5, 10, 25, 50, and 100 μM) under photoirradiation/non-irradiation conditions. The cell viability is presented as mean ± s.d. (*n* = 3 independent experiments). Source data are provided as a Source Data file.

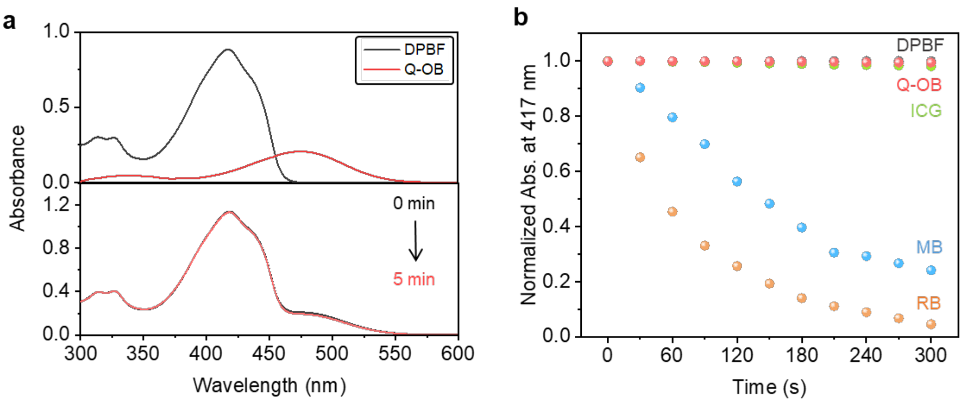

Supplementary Figure 45. Characterization of phototoxicity of Q-OB. **a** Absorption spectra of 1,3-diphenylisobenzofuran (DPBF) (50 μM) and Q-OB (5 μM), and irradiation time-dependent UV/Vis spectrum change of DPBF in the presence of Q-OB in DMSO under light irradiation of Xe-lamp up to 5 min (λ_{ex} = 488 nm, 1 mW/cm^2). **b** Absorbance changes of DPBF at 417 nm using Q-OB, indocyanine green (ICG), methylene blue (MB), and rose bengal (RB) upon photoirradiation (λ_{ex} = 488 (Q-OB), 530 (RB), 660 (MB), 800 (ICG) nm, respectively) (photosensitizers conc. = 5 μM). Source data are provided as a Source Data file.

Supplementary Table 5. Comparison of Q-OB properties with blood-brain barrier (BBB) penetrability selection rules.

Selection rules	Ideal ranges	Q-OB
Topological polar surface area (TPSA)[a]	< 90 Å^2	36.53 Å^2
Number of atoms	20 to 70	27
H-bond donors	< 5	0
H-bond acceptors	< 10	4
Rotatable bond	< 10	5
Calculated log P[b]	0.9 to 5.6	5.13 ± 1.16
Calculated log D[c]	0.9 to 5.6	5.05 ± 1.14
Molecular weight	180 to 480	372.2166

[a]Calculated using the Molinspiration cheminformatics applet (https://www.molinspiration.com/), and [b]calculated from pooled log P data, as implemented by the ALOGPS 2.1 applet (http://www.vcclab.org/lab/alogps/). [c]Q-OB is neutral at physiological pH (see Supplementary Figure 27), thus log D ≈ log P.

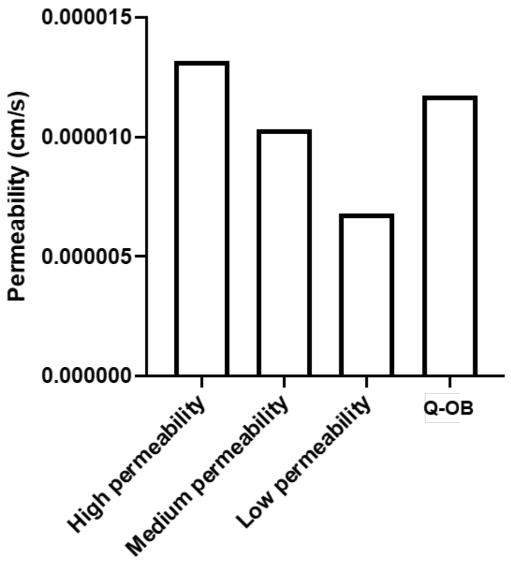

Supplementary Figure 46. Parallel artificial membrane permeability assay (PAMPA) BBB penetrability assay of Q-OB compared to reference compounds.

Supplementary Table 6. Effective permeability (P_e) value of PAMPA-BBB penetrability assay of Q-OB compared to reference compounds.

Compound	P_e (cm/s)	–log P_e	CNS prediction
High permeability	1.3184E-05	4.87996275	CNS+
Medium permeability	1.0312E-05	4.98664709	CNS+
Low permeability	6.7983E-06	5.16760096	CNS±
Q-OB	1.1706E-05	4.93158396	CNS+

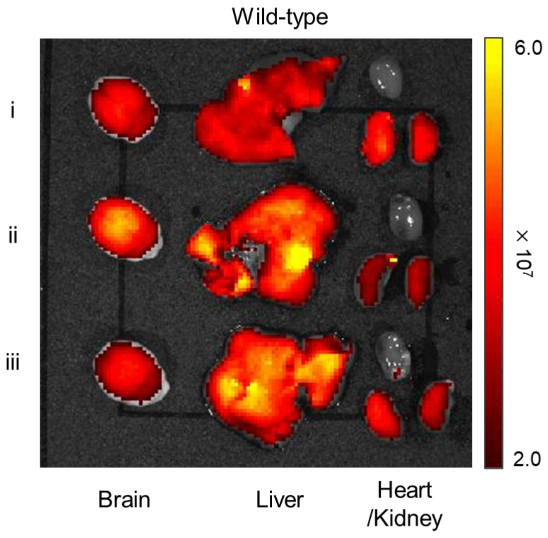

Supplementary Figure 47. Ex vivo fluorescence imaging of major organs (brain, liver, heart, and kidney) of 2-month-old B6 Wild-type mice ($n = 3$ biologically independent animals). The fluorescence images were captured using excitation at 500 nm, and the emission was monitored at 620 nm.

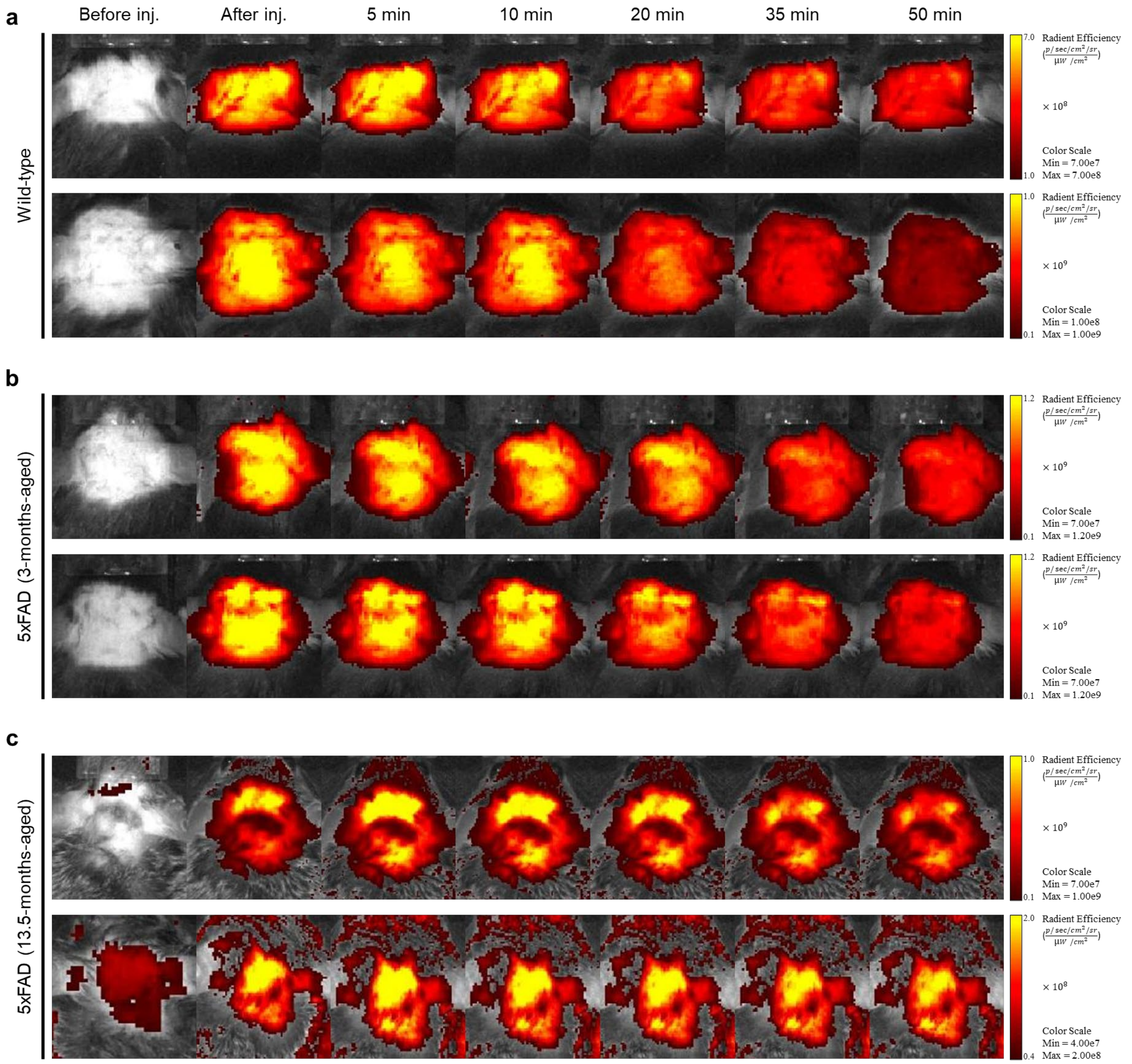

Supplementary Figure 48. In vivo fluorescence imaging of (**a**) 2-month-old B6 Wild-type (*n* = 3 biologically independent animals), (**b**) 3-month-old 5xFAD (*n* = 3 biologically independent animals), and (**c**) 13.5-month-old 5xFAD (*n* = 3 biologically independent animals) transgenic AD model mice brains. The fluorescence signal of in vivo imaging, 50 min after tail vein injection of Q-OB. The fluorescence images were collected using excitation at 500 nm, and the emission was monitored at 620 nm.

Supplementary Table 7. Clinical data and sample information of patients' cerebrospinal fluid (CSF) biomarkers in AD continuum.

No	Diagnosis	Object ID	Sex	APOE	PET	SUVR	Q-OB I/I_0	Q-OB Log (I/I_0)	ELISA_CSF $A\beta_{1-42}$	Procartaplex_ CSF p-Tau_{181}	ELISA_CSF NFL
1	CN	1456	F	3/3	Negative	1.04	1.69	0.228	1247.4	6.29	623.3
2	CN	1482	M	3/4	Negative	0.98	2.26	0.355	1206.2	8.75	448.0
3	CN	3346	M	2/3	Negative	1.09	1.59	0.202	1055.7	9.30	561.8
4	CN	4192	M	3/4	Negative	0.94	2.06	0.315	1197.4	7.95	784.5
5	CN	5192	M	3/3	Negative	1.01	1.92	0.283	1253.8	8.56	447.9
6	CN	5300	F	2/3	Negative	1.00	1.73	0.237	1180.2	11.74	739.4
7	CN	5838	F	3/3	Negative	0.99	1.78	0.250	1064.5	8.38	518.5
8	CN	6327	M	2/2	Negative	0.94	1.83	0.262	1012.4	8.43	330.5
9	CN	6428	F	3/3	Negative	0.96	2.78	0.444	1243.6	9.01	420.1
10	CN	6509	F	3/3	Negative	0.98	2.09	0.319	1597.0	16.81	407.3
11	CN	6161	M	3/3	Negative	0.98	1.97	0.294	1123.0	15.59	1196.8
12	CN	6751	M	3/3	Negative	0.92	1.98	0.297	1268.3	12.21	764.9
13	CN	2898	M	3/3	Negative	0.93	1.99	0.299	1390.4	8.00	315.7
14	CN	6010	F	3/3	Negative	0.99	1.53	0.184	1295.1	6.03	354.7
15	CN	6160	F	3/3	Negative	0.98	2.52	0.401	1082.5	5.66	289.2
16	CN	5486	F	3/3	Negative	1.08	2.45	0.388	1445.2	8.58	617.7
17	CN	5441	F	3/4	Negative	1.04	5.22	0.718	1190.7	9.07	783.9
18	CN	3385	M	3/3	Negative	0.99	3.42	0.534	1402.3	13.35	528.2
19	CN	5675	F	3/3	Negative	0.98	2.92	0.466	1033.8	13.47	740.7
20	CN	6425	F	3/3	Negative	0.99	2.81	0.449	1058.2	11.17	658.8
21	CN	3946	F	3/3	Negative	0.98	2.27	0.356	1303.0	9.71	683.6
22	CN	8180	M	3/4	Negative	0.90	1.41	0.149	1136.1	8.50	446.0
23	MCI	91	M	3/4	Positive	1.44	2.23	0.348	374.5	14.02	304.0
24	MCI	174	M	3/3	Positive	1.25	1.23	0.091	452.9	35.00	1043.1
25	MCI	2043	F	3/3	Positive	1.49	1.15	0.059	342.1	18.15	570.2
26	MCI	2952	M	2/3	Positive	1.49	2.10	0.323	280.5	31.13	631.9
27	MCI	5246	M	3/4	Positive	1.41	1.54	0.187	152.7	20.18	637.3
28	MCI	5979	M	3/4	Positive	1.29	2.95	0.470	487.3	6.77	460.5
29	MCI	6512	M	3/4	Positive	1.31	1.22	0.086	514.8	23.50	1005.2
30	MCI	6930	F	4/4	Positive	1.34	1.15	0.060	354.3	28.33	385.3
31	MCI	7043	F	3/3	Positive	1.20	1.29	0.112	352.2	36.21	2112.1
32	MCI	7321	M	3/3	Positive	1.46	1.41	0.149	271.0	23.30	633.4
33	MCI	7645	M	3/3	Positive	1.33	1.10	0.042	329.3	17.36	455.6
34	MCI	7730	M	3/4	Positive	1.54	1.18	0.073	198.5	44.58	978.0
35	MCI	7997	F	4/4	Positive	1.58	1.81	0.257	166.9	46.51	755.9
36	MCI	8105	F	4/4	Positive	1.53	1.24	0.093	220.6	29.46	546.6
37	MCI	8143	F	2/3	Positive	1.59	1.30	0.115	563.7	36.69	979.7

38	MCI	8152	M	3/3	Positive	1.55	1.50	0.177	459.7	28.76	800.5
39	MCI	10587	F	3/4	Positive	1.31	1.83	0.262	458.0	16.94	1118.9
40	MCI	10863	F	3/4	Positive	1.34	1.18	0.073	499.3	19.93	562.8
41	MCI	11245	F	4/4	Positive	1.20	1.36	0.135	465.8	30.54	592.4
42	MCI	12711	F	3/4	Positive	1.47	1.48	0.172	408.5	18.02	489.3
43	MCI	13720	M	3/4	Positive	n.d.	1.01	0.005	467.5	13.01	428.0
44	ADD	3205	M	3/3	Positive	1.40	2.64	0.422	335.8	19.37	798.9
45	ADD	4036	M	3/4	Positive	1.36	1.25	0.095	412.2	18.77	809.1
46	ADD	5577	M	4/4	Positive	1.28	1.29	0.111	364.0	15.50	1174.6
47	ADD	5621	F	3/4	Positive	1.37	1.33	0.123	468.2	16.38	679.3
48	ADD	5716	F	3/4	Positive	1.48	1.31	0.116	443.4	24.38	797.6
49	ADD	5945	F	3/4	Positive	1.48	1.33	0.123	311.5	25.72	1926.0
50	ADD	5961	F	3/3	Positive	1.37	1.17	0.070	422.8	18.34	741.9
51	ADD	6005	F	2/3	Positive	1.55	1.73	0.239	382.5	18.83	883.3
52	ADD	6216	M	3/3	Positive	1.43	1.20	0.078	527.1	36.29	870.5
53	ADD	7239	F	3/4	Positive	1.33	1.14	0.055	495.5	29.60	1204.0
54	ADD	7627	F	3/3	Positive	1.45	1.33	0.125	373.9	25.73	1259.5
55	ADD	6670	F	3/4	Positive	1.42	1.05	0.021	376.9	18.63	791.1
56	ADD	6979	M	3/4	Positive	1.49	0.98	-0.007	413.9	25.47	1741.7
57	ADD	12997	F	3/4	Positive	1.2	1.21	0.083	471.5	21.03	1030.2
58	ADD	6808	F	2/4	Positive	1.43	1.54	0.189	355.0	17.56	511.5
59	ADD	9761	F	3/4	Positive	1.49	1.73	0.238	346.1	13.49	499.3
60	ADD	8354	M	3/3	Positive	1.46	1.47	0.166	198.7	22.25	1212.6
61	ADD	12813	M	3/4	Positive	1.14	1.32	0.122	159.7	12.45	1034.6

Written informed consent was obtained from each participant or their legal guardian. Object ID was designated by Gwangju Alzheimer's Disease and Related Dementias Cohort Center). The abbreviated words are apolipoprotein E (APOE), positron emission tomography (PET), standardized uptake value ratio (SUVR), enzyme-linked immunosorbent assay (ELISA), amyloid-β (Aβ), cerebrospinal fluids (CSF), phosphorylated tau (p-Tau), neurofilament light chain (NFL), Cognitive normal (CN), Mild cognitive impairment (MCI), Alzheimer's disease dementia (ADD), Female (F), Male (M). Not available data was marked as n.d. Source data are provided as a Source Data file.

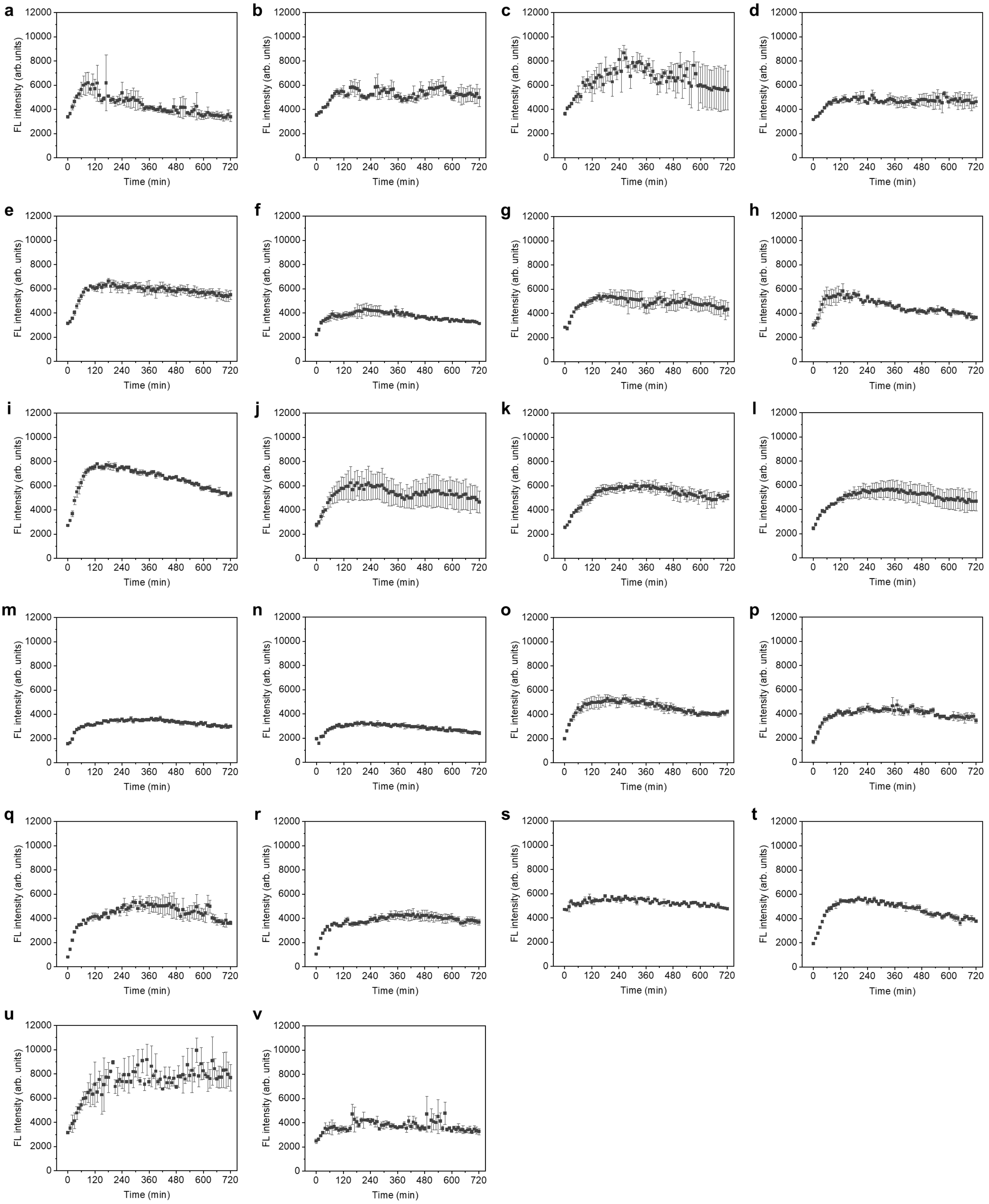

Supplementary Figure 49. In vitro Q-OB assay results for early AD diagnosis using patients' CSF samples. Time-dependent fluorescence intensity change of Q-OB (1 μM)-treated cognitive normal (CN) CSF samples during coincubation (37°C, 200 rpm, for 12 h) with exogenously added A$\beta_{1\text{–}42}$ monomer (10 μM) from sample number 1 to 22 in Supplementary Table 7 (**a** to **v**, respectively). Data are represented as mean ± s.d. with n = 22 individually independent samples examined over 3 independent measurements. Source data are provided as a Source Data file.

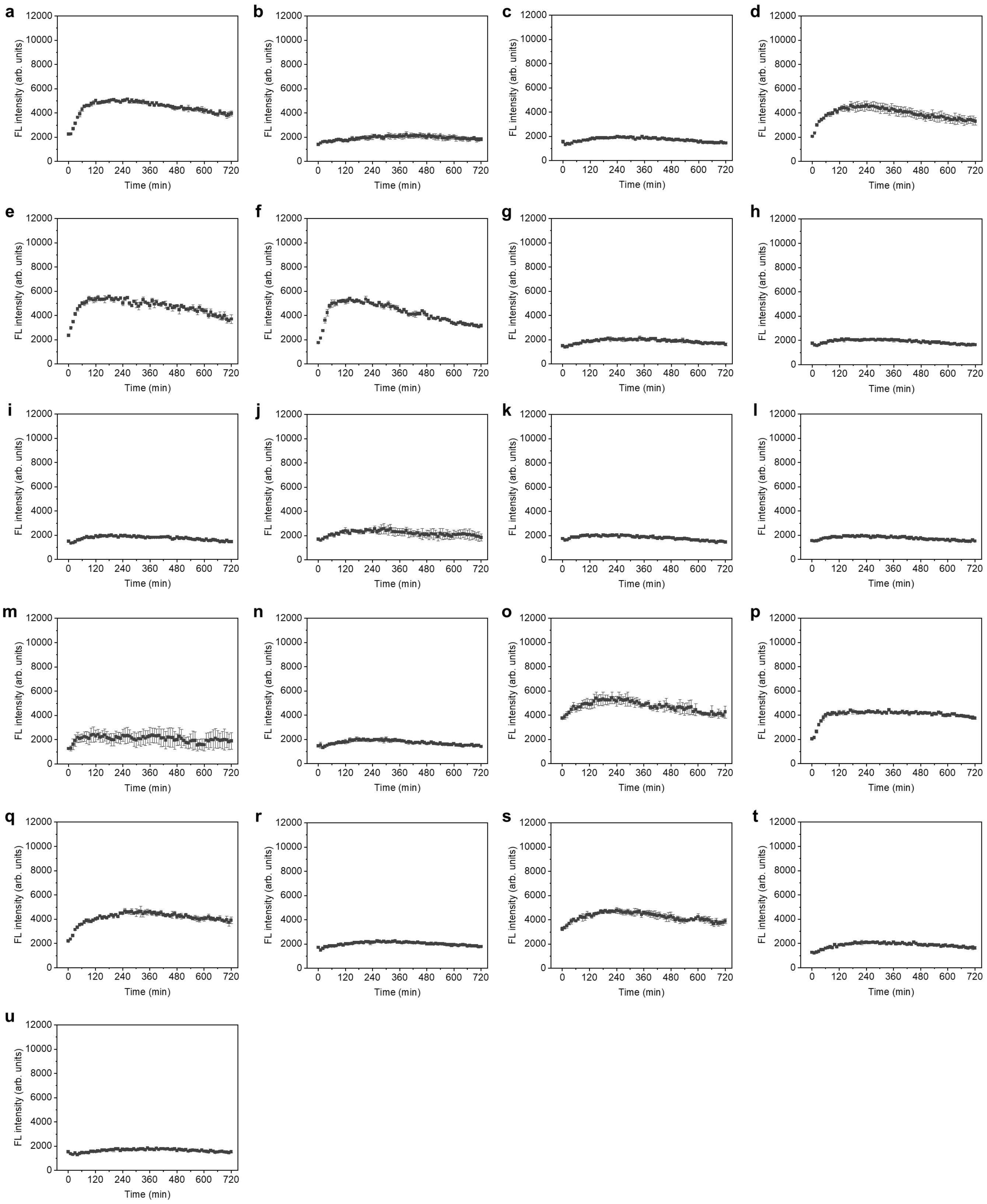

Supplementary Figure 50. In vitro Q-OB assay results for early AD diagnosis using patients' CSF samples. Time-dependent fluorescence intensity change of Q-OB (1 μM)-treated mild cognitive impairment (MCI) CSF samples during coincubation (37°C, 200 rpm, for 12 h) with exogenously added $A\beta_{1-42}$ monomer (10 μM) from sample number 23 to 43 in Supplementary Table 7 (**a** to **u**, respectively). Data are represented as mean ± s.d. with n = 21 individually independent samples examined over 3 independent measurements. Source data are provided as a Source Data file.

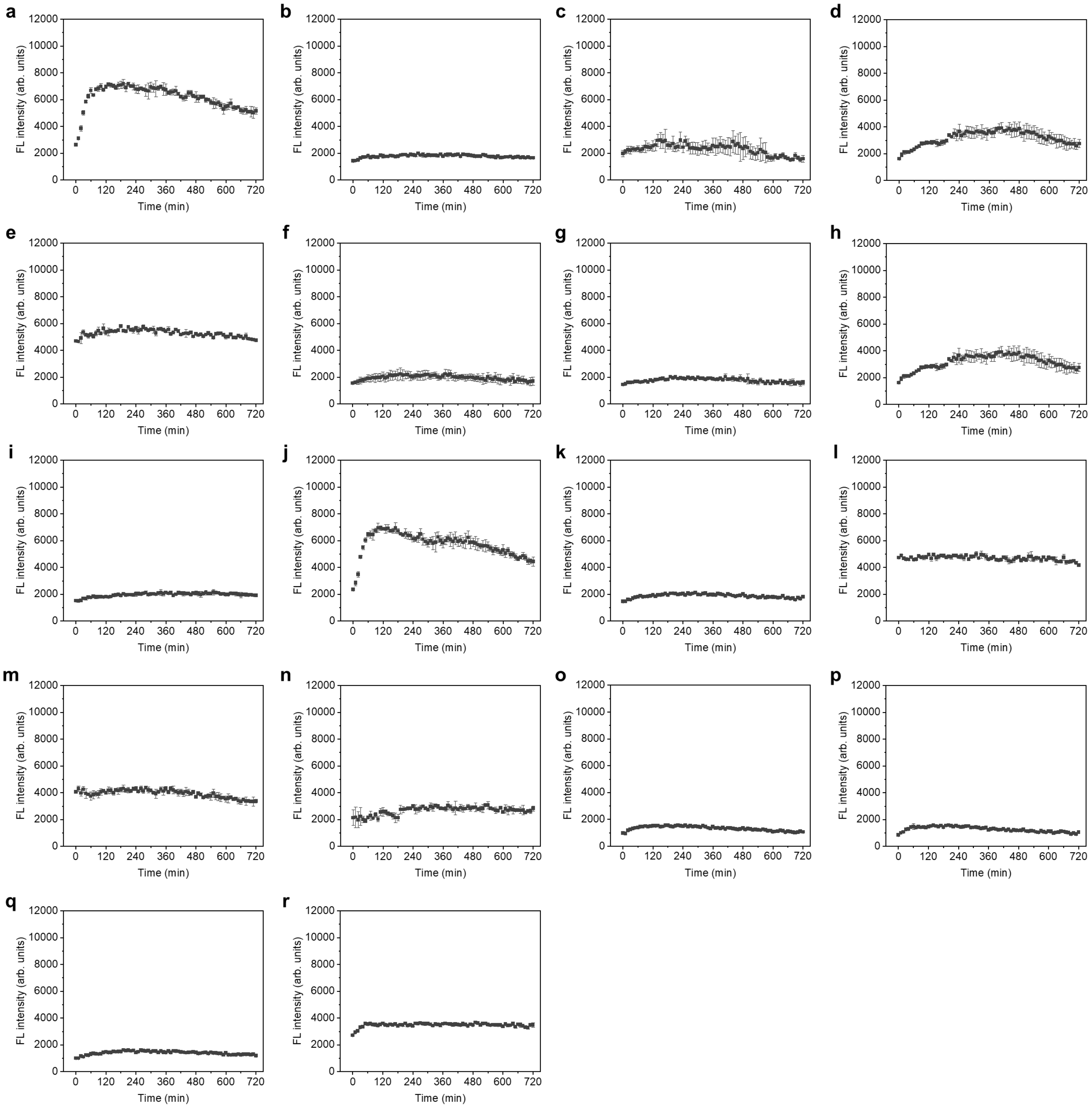

Supplementary Figure 51. In vitro Q-OB assay results for early AD diagnosis using patients' CSF samples. Time-dependent fluorescence intensity change of Q-OB (1 μM)-treated AD dementia (ADD) CSF samples during coincubation (37°C, 200 rpm, for 12 h) with exogenously added $A\beta_{1\text{–}42}$ monomer (10 μM) from sample number 44 to 61 in Supplementary Table 7 (**a** to **r**, respectively). Data are represented as mean ± s.d. with n = 18 individually independent samples examined over 3 independent measurements. Source data are provided as a Source Data file.

Supplementary References

1 Drake, J. M., Lesiecki, M. L. & Camaioni, D. M. Photophysics and cis-trans isomerization of DCM. *Chem. Phys. Lett.* **113**, 530–534, doi:https://doi.org/10.1016/0009-2614(85)85026-0 (1985).

2 Brouwer, A. M. Standards for photoluminescence quantum yield measurements in solution (IUPAC Technical Report). **83**, 2213–2228, doi:doi:10.1351/PAC-REP-10-09-31 (2011).

3 Gaussian 16 Rev. C.01 (Wallingford, CT, 2016).

4 Chai, J.-D. & Head-Gordon, M. Long-range corrected hybrid density functionals with damped atom–atom dispersion corrections. *Phys. Chem. Chem. Phys.* **10**, 6615–6620, doi:10.1039/B810189B (2008).

5 Barone, V., Cimino, P. & Stendardo, E. Development and Validation of the B3LYP/N07D Computational Model for Structural Parameter and Magnetic Tensors of Large Free Radicals. *J. Chem. Theory Comput.* **4**, 751–764, doi:10.1021/ct800034c (2008).

6 Le Bahers, T., Adamo, C. & Ciofini, I. A Qualitative Index of Spatial Extent in Charge-Transfer Excitations. *J. Chem. Theory Comput.* **7**, 2498–2506, doi:10.1021/ct200308m (2011).

7 Lu, T. & Chen, F. Multiwfn: A multifunctional wavefunction analyzer. *J. Comput. Chem.* **33**, 580–592, doi:https://doi.org/10.1002/jcc.22885 (2012).

8 Yanai, T., Tew, D. P. & Handy, N. C. A new hybrid exchange–correlation functional using the Coulomb-attenuating method (CAM-B3LYP). *Chem. Phys. Lett.* **393**, 51–57, doi:https://doi.org/10.1016/j.cplett.2004.06.011 (2004).

9 Teoh, C. L. *et al.* Chemical Fluorescent Probe for Detection of Aβ Oligomers. *J. Am. Chem. Soc.* **137**, 13503–13509, doi:10.1021/jacs.5b06190 (2015).

10 Zhang, X. *et al.* Near-infrared fluorescence molecular imaging of amyloid beta species and monitoring therapy in animal models of Alzheimer's disease. *Proc. Natl. Acad. Sci. U. S. A.* **112**, 9734–9739, doi:10.1073/pnas.1505420112 (2015).

11 Zhang, X. *et al.* Design and Synthesis of Curcumin Analogues for in Vivo Fluorescence Imaging and Inhibiting Copper-Induced Cross-Linking of Amyloid Beta Species in Alzheimer's Disease. *J. Am. Chem. Soc.* **135**, 16397–16409, doi:10.1021/ja405239v (2013).

12 Li, Y. *et al.* Tuning the stereo-hindrance of a curcumin scaffold for the selective imaging of the soluble forms of amyloid beta species. *Chem. Sci.* **8**, 7710–7717, doi:10.1039/C7SC02050C (2017).

13 Yang, J. *et al.* Half-curcumin analogues as PET imaging probes for amyloid beta species. *Chem. Commun.* **55**, 3630–3633, doi:10.1039/C8CC10166C (2019).

14 Yang, J. *et al.* Highly specific detection of Aβ oligomers in early Alzheimer's disease by a near-infrared fluorescent probe with a "V-shaped" spatial conformation. *Chem. Commun.* **56**, 583–586, doi:10.1039/C9CC08894F (2020).

15 Ge, Y. *et al.* Curcumin Complex Analogues as Near-Infrared Fluorescent Probes for Monitoring all Aβ Species in the Early Alzheimer's Disease Model. *ACS Chem. Neurosci.* **12**, 3683–3689, doi:10.1021/acschemneuro.1c00419 (2021).

16 Lv, G. *et al.* A spiropyran-based fluorescent probe for the specific detection of β-amyloid peptide oligomers in Alzheimer's disease. *Chem. Commun.* **52**, 8865–8868, doi:10.1039/C6CC02741E (2016).

17 Li, Y. *et al.* Fluoro-substituted cyanine for reliable in vivo labelling of amyloid-β oligomers and neuroprotection against amyloid-β induced toxicity. *Chem. Sci.* **8**, 8279–8284, doi:10.1039/C7SC03974C (2017).

18 Lv, G. *et al.* A novel near-infrared fluorescent probe for detection of early-stage Aβ protofibrils in Alzheimer's disease. *Chem. Commun.* **56**, 1625–1628, doi:10.1039/C9CC09233A (2020).

19 Li, H. *et al.* Detection of Aβ oligomers in early Alzheimer's disease diagnose by in vivo NIR-II fluorescence imaging. *Sens. Actuator B-Chem.* **358**, 131481, doi:https://doi.org/10.1016/j.snb.2022.131481 (2022).

20 Jameson, L. P. & Dzyuba, S. V. Aza-BODIPY: Improved synthesis and interaction with soluble Aβ1–42 oligomers. *Bioorg. Med. Chem. Lett.* **23**, 1732–1735, doi:https://doi.org/10.1016/j.bmcl.2013.01.065 (2013).

21 Sun, L. *et al.* Amphiphilic Distyrylbenzene Derivatives as Potential Therapeutic and Imaging Agents for Soluble and

Insoluble Amyloid β Aggregates in Alzheimer's Disease. *J. Am. Chem. Soc.* **143**, 10462–10476, doi:10.1021/jacs.1c05470 (2021).

22 Lee, D., Kim, S. M., Kim, H. Y. & Kim, Y. Fluorescence Chemicals To Detect Insoluble and Soluble Amyloid-β Aggregates. *ACS Chem. Neurosci.* **10**, 2647–2657, doi:10.1021/acschemneuro.9b00199 (2019).

23 An, J. *et al.* Picomolar-sensitive β-amyloid fibril fluorophores by tailoring the hydrophobicity of biannulated π-elongated dioxaborine-dyes. *Bioact. Mater.* **13**, 239–248, doi:https://doi.org/10.1016/j.bioactmat.2021.10.047 (2022).

24 Cho, I. *et al.* Immobilized Amyloid Hexamer Fragments to Map Active Sites of Amyloid-Targeting Chemicals. *ACS Chem. Neurosci.* **14**, 9–18, doi:10.1021/acschemneuro.2c00449 (2023).
