# Supplementary material for: Early onset diagnosis in Alzheimer’s disease patients via amyloid-β oligomers-sensing probe in cerebrospinal fluid
Source: Nat Commun. 2024 Feb 2;15:1004. doi: 10.1038/s41467-024-44818-x (PMC10837422; doi:10.1038/s41467-024-44818-x)
Supplement: Supplementary file 3 — Reporting Summary [file 41467_2024_44818_MOESM3_ESM.pdf]

nature portfolio

Corresponding author(s): YoungSoo Kim, Kun Ho Lee, Jong Seung Kim

Last updated by author(s): Dec 14, 2023

Reporting Summary

Nature Portfolio wishes to improve the reproducibility of the work that we publish. This form provides structure for consistency and transparency in reporting. For further information on Nature Portfolio policies, see our Editorial Policies and the Editorial Policy Checklist.

Statistics

For all statistical analyses, confirm that the following items are present in the figure legend, table legend, main text, or Methods section.

n/a	Confirmed	
☐	☒	The exact sample size (*n*) for each experimental group/condition, given as a discrete number and unit of measurement
☐	☒	A statement on whether measurements were taken from distinct samples or whether the same sample was measured repeatedly
☐	☒	The statistical test(s) used AND whether they are one- or two-sided<br>*Only common tests should be described solely by name; describe more complex techniques in the Methods section.*
☐	☒	A description of all covariates tested
☐	☒	A description of any assumptions or corrections, such as tests of normality and adjustment for multiple comparisons
☐	☒	A full description of the statistical parameters including central tendency (e.g. means) or other basic estimates (e.g. regression coefficient) AND variation (e.g. standard deviation) or associated estimates of uncertainty (e.g. confidence intervals)
☐	☒	For null hypothesis testing, the test statistic (e.g. *F*, *t*, *r*) with confidence intervals, effect sizes, degrees of freedom and *P* value noted<br>*Give P values as exact values whenever suitable.*
☒	☐	For Bayesian analysis, information on the choice of priors and Markov chain Monte Carlo settings
☒	☐	For hierarchical and complex designs, identification of the appropriate level for tests and full reporting of outcomes
☒	☐	Estimates of effect sizes (e.g. Cohen's *d*, Pearson's *r*), indicating how they were calculated

Our web collection on statistics for biologists contains articles on many of the points above.

Software and code

Policy information about availability of computer code

Data collection	Spectroscopy Source Data were collected using Bruker NMR spectrometer (500 MHz) (USA), Jasco V-750 spectrometer, Jasco FP-8500 spectrofluorometer, Jasco J-1500 spectrometer, and Hidex Sense Microplate reader with commercial software provided by the suppliers. Imaging data were collected on IVIS spectrum (Perkin Elmer, USA). No customized software was used for data collection in this study.
Data analysis	Spectroscopy Source Data were analyzed using OriginPro 2020 (64-bit) (OriginLab Corporation, ver. 9.7.0.185 (Academic)) and MestReNova (Mestrelab Research S.L., ver. 6.0.2-5475). Imaging data were analyzed with IVIS Living-Imaging software, associated with the IVIS Spectrum imaging system, and ImageJ.

For manuscripts utilizing custom algorithms or software that are central to the research but not yet described in published literature, software must be made available to editors and reviewers. We strongly encourage code deposition in a community repository (e.g. GitHub). See the Nature Portfolio guidelines for submitting code & software for further information.

Data

Policy information about availability of data

All manuscripts must include a data availability statement. This statement should provide the following information, where applicable:

- Accession codes, unique identifiers, or web links for publicly available datasets
- A description of any restrictions on data availability
- For clinical datasets or third party data, please ensure that the statement adheres to our policy

All data supporting the finding of this study are included in the main article and its Supplementary Information. All Source Data are available in publicly accessible

repository or from the corresponding author upon reasonable request.

Research involving human participants, their data, or biological material

Policy information about studies with human participants or human data. See also policy information about sex, gender (identity/presentation), and sexual orientation and race, ethnicity and racism.

Reporting on sex and gender	Participants' sex was included in this study and reported in Supplementary Tables 4 and 5 (Supplementary Information) and its Source Data. Written informed consent was obtained from each participant or their legal guardian. Sex-based analysis was not performed as this was not part of the objectives of this study.
Reporting on race, ethnicity, or other socially relevant groupings	We did not use any socially constructed or socially relevant groupings.
Population characteristics	For histochemical analysis of human brain tissues, postmortem cerebral hippocampal tissue specimens were recieved from the Victorian Brain Bank Network (VBBN) without clinical information. Participant charcteristics in this study include sex (male or female), age (at the time of postmortem examination and collected a non-specific age for this study), type of disease stage (cognitive normal or Alzheimer's disease dementia), post-mortem interval (PMI, hour), and apolipoprotein E genetic types in Supplementary Table 4 (Supplementary Information).<br>For cerebrospinal fluid analysis, cerebrospinal fluids and all clinical data were collected in the Gwangju Alzheimer's Disease and Related Dementias Cohort Center. Participant characteristics in this study include sex (male or female), age (at the time of sample collection and collected a non-specific age for this study), type of disease stage (cognitive normal, mild cognitive impairment, or Alzheimer's disease dementia), apolipoprotein E genetic types, positron emission tomography (standardized uptake value ratio, SUVR), neuropsychological domain (using the Seoul Neuropsychological Screening Battery (SNSB), and analytical results of Lumipulse fully automated immunoassay and two manual immunoassays (INNOBIA AlzhBio3 xMAP, INNOTEST). All participants recieved no prior treatment.
Recruitment	This study included Alzheimer's disease dementia patients and healthy (non-demented) participants who volunteered for a medical examination at the Gwangju Alzheimer's Disease and Related Dementias Cohort Center. Cerebrospinal fluid samples of each participant were obtained according to the preference of each participant or their legal guardian. The preference resulted from consideration of spinal cord injection, and the participants' self-selection bias or other biases arising during this sampling process did not affect the results of this study.
Ethics oversight	This study was approved by Victorian Institute of Forensic Medicine (VIFM) Ethics Committee and Human Ethics Committee STEMM 1 (Ref. No. 2020-20326-13293-3) of the University of Melbourne and the Institutional Review Boards of Chosun University Hospital (CHOSUN 2013-12-018-068) and Chonnam National University Hospital (CNUH-2019-279) (Republic of Korea). Written informed consents were obtained from each participant or their legal guardian.

Note that full information on the approval of the study protocol must also be provided in the manuscript.

Field-specific reporting

Please select the one below that is the best fit for your research. If you are not sure, read the appropriate sections before making your selection.

[x] Life sciences [ ] Behavioural & social sciences [ ] Ecological, evolutionary & environmental sciences

For a reference copy of the document with all sections, see nature.com/documents/nr-reporting-summary-flat.pdf

Life sciences study design

All studies must disclose on these points even when the disclosure is negative.

Sample size	No sample-size calculation was performed. Sufficient spectroscopy results were collected from experience to ensure their representation of the sample. All experiments were performed with at least n = 3 independently prepared samples for statistical tests. NMR spectroscopy, Mass spectroscopy, and fundamental photophysical properties (including absorption wavelength, fluorescence emission wavelength, and fluorescence quantum yield analysis), computational calculation which only instrumental/computational errors are considered were performed once (n = 1). Even though we did not set up the sample size using the sample-size calculation, the data were obtained as well-reproductive results even in the independent condition (experimental data; interval: about 1 month).
Data exclusions	No exclusion of data was involved for in vitro experiments. For in vivo mouse studies, data was excluded if the injection failed.
Replication	All experiments are replicable according to the described procedures in the manuscript and Supplementary Information. Unless explicitly stated, all data shown were obtained from at least three independent experiments. For representative images, each experiment was successfully repeated at least three times under similar conditions independently.
Randomization	No randomization was required for in vitro samples and in vivo organisms described in manuscript and Supplementary Information. Randomization of experimental groups is not relevant to these study for detecting analytes and imaging method validation. Only the order of fluorometric analysis using cerebrospinal fluid samples for disease diagnosis was randomized to the experimentalist.
Blinding	No blinding study was performed for in vitro experiments and in vivo imaging described in manuscript and Supplementary Information. For these experiments, we must mark the group name, count of analytes, and experiment date on each samples because we have been thoroughly monitored under Ethics Council. We alternatively performed the reproductive analysis though we did not consider the blind test.

Only the written information of participants who provided cerebrospinal fluid samples for examination (disease diagnosis) through fluorometric analysis was blinded to the experimentalist during measurements before data analysis.

Reporting for specific materials, systems and methods

We require information from authors about some types of materials, experimental systems and methods used in many studies. Here, indicate whether each material, system or method listed is relevant to your study. If you are not sure if a list item applies to your research, read the appropriate section before selecting a response.

Materials & experimental systems

n/a	Involved in the study	
☐	☒	Antibodies
☐	☒	Eukaryotic cell lines
☒	☐	Palaeontology and archaeology
☐	☒	Animals and other organisms
☒	☐	Clinical data
☒	☐	Dual use research of concern
☒	☐	Plants

Methods

n/a	Involved in the study	
☒	☐	ChIP-seq
☒	☐	Flow cytometry
☒	☐	MRI-based neuroimaging

Antibodies

Antibodies used	For histological staining of mice and human brain tissues, anti-amyloid-β monoclonal antibody 6E10 (amyloid-β1-16 antibody, 1:200,Biolegend, USA, #SIG-39320), 4G8 (amyloid-β17-24, 1:200, Biolegend, USA, #800701) were used as a primary antibody and anti-mouse IgG-Alexa 350 (1:200, Invitrogen, USA, #A11045) was used as a secondary antibody. A commercially available analytical platform, Lumipulse fully automated immunoassay and two manual immunoassays (INNOBIA AlzhBio3 xMAP, INNOTEST) kit with research-only reagents (Fujirebio, Tokyo, Japan, #80584), was used according to the manufacturer's protocols.
Validation	Vaidation of primary antibodies 6E10 and 4G8 were summerized from manufacturer's protocols (6E10: https://www.biolegend.com/ja-jp/cellular-dyes-and-ancillary-products/purified-anti-beta-amyloid-1-16-antibody-11228; 4G8: https://www.biolegend.com/en-gb/search-results/purified-anti-beta-amyloid-17-24-antibody-11233?GroupID=BLG15648). Validation of secondary antibody anti-mouse IgG-Alexa 350 was summerized from manufacturer's protocols (anti-mouse IgG-Alexa 350: https://www.thermofisher.com/antibody/product/Goat-anti-Mouse-IgG-H-L-Cross-Adsorbed-Secondary-Antibody-Polyclonal/A-11045).<br>Regarding INNOBIA AlzBio3 xMAP kit, the absolute concentration of Aβ1-42, phosphorylated tau181, and neurofilament light chain in cerebrospinal fluids were measured according to the manufacturer's protocols (https://www.launchdiagnostics.com/product/inno-bia-alzbio3-fruo-2/).

Eukaryotic cell lines

Policy information about cell lines and Sex and Gender in Research

Cell line source(s)	Human-derived neuroblastoma cell line SH-SY5Y was purchased from The American Type Culture Collection (ATCC) (https://www.atcc.org/products/crl-2266).
Authentication	We purchased the SK-N-SH (ATCC HTB-11) cell line (SH-SY5Y) that is certified by the American Type Culture Collection (ATCC) without additional authentication service.
Mycoplasma contamination	We declare that the cell line was not test for mycoplasma contamination.
Commonly misidentified lines (See ICLAC register)	We did not used the misidentified cell lines in this work.

Animals and other research organisms

Policy information about studies involving animals; ARRIVE guidelines recommended for reporting animal research, and Sex and Gender in Research

Laboratory animals	2- and 8-month-old B6 wild-type; 3-, 12-, 13.5, and 16-month-old 5xFAD transgenic AD model mice of either sex. Laboratory mice generally are maintained at macroenvironmental temperature and humidity ranges of 65 to 75°F (18 to 23°C) and 40% to 60%, respectively.
Wild animals	No wild animals were used in this study.
Reporting on sex	Sex was not considered in this study design or methods.
Field-collected samples	No field-collected samples were used in this study.
Ethics oversight	All animal experiments and procedures were approved by the Committee for the Care and Use of Laboratory Animals at Yonsei

University (Republic of Korea, IACUC-A-202205-1479-01).

Note that full information on the approval of the study protocol must also be provided in the manuscript.

Plants

Seed stocks	n/a
Novel plant genotypes	n/a
Authentication	n/a
